# Catalytic conversion of methane to methanol: recent advances in MOF-based catalysts, oxidant strategies, and process optimization

**DOI:** 10.1039/d6ra01479h

**Published:** 2026-07-02

**Authors:** U. Zayyanu Gwandu, G. Abdulkareem-Alsultan, G. Mohammad-Alsultan, N. Asikin-Mijan, N. Asma-Samsudin, H. V. Lee, Yun Hin Taufiq-Yap

**Affiliations:** a Catalysis Science and Technology Research Centre (PutraCat), Faculty of Science, Universiti Putra Malaysia 43400 UPM Serdang Selangor Malaysia kreem.alsultan@yahoo.com taufiq@upm.edu.my; b Department of Chemical Sciences, Faculty of Science and Technology, Universiti Kebangsaan Malaysia, UKM Bangi Selangor Darul Ehsan 43600 Malaysia nurul.asikin@ukm.edu.my; c Nanotechnology and Catalysis Research Centre (NanoCat), Institute of Postgraduate Studies, University Malaya 50603 Kuala Lumpur Malaysia; d Fuel and Energy Techniques Eng. Department, South Technical University Al-Zubair Street Basra Iraq; e Department of Petroleum Project Management College of Industrial Management of Oil and Gas, Basrah University for Oil and Gas Basra Iraq

## Abstract

The conversion of methane to methanol through selective oxidation has attracted considerable attention as an eco-friendly method for utilizing natural gas and reducing greenhouse gas emissions. This review offers an in-depth examination of catalytic methods for methane oxidation, with special emphasis on the latest developments relating to catalysts based on metal–organic frameworks (MOFs). The discussion covers the function of MOFs as catalysts, their structural benefits, and the integration of nanoparticles (NPs), including both monometallic and bimetallic NP@MOF systems, to boost catalytic performance. Additionally, traditional catalysts such as supported monometallic and bimetallic catalysts, which include noble and transition metals, are evaluated for their effectiveness in oxidizing methane. The review also investigates various oxidants used in the conversion of methane to methanol, focusing on oxygen-based systems. Moreover, different reactor setups used for methane oxidation, especially hydrothermal reactors, are assessed for their operational efficiency. Parameters for optimization, such as reaction time, temperature, pressure, pH, and water content, are discussed to provide insights into enhancing methanol selectivity and yield. By incorporating recent progress relating to catalytic materials, reactor design, and optimization strategies, this review aims to present a thorough perspective on the current status and future prospects of methane-to-methanol conversion technologies.

## Abstract 2

The conversion of methane to methanol through selective oxidation has attracted considerable attention as an eco-friendly method for utilizing natural gas and reducing greenhouse gas emissions.

## Graphical abstract

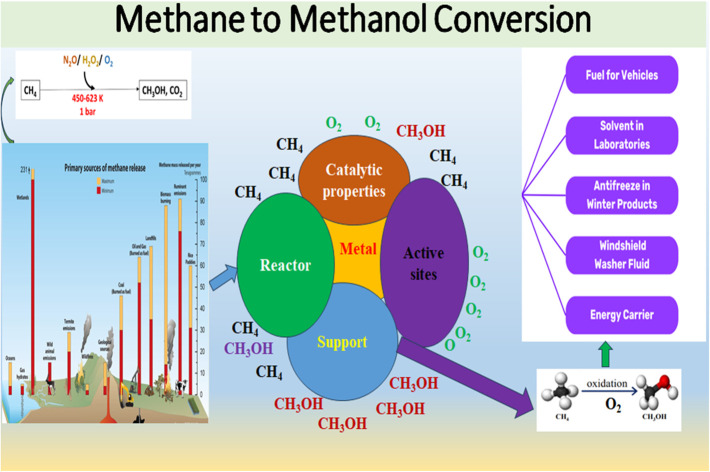

## 1 Introduction

Methane is the major component of natural gas and a significant component of landfill gas (40–60 c\vol%), biogas and cold mine drainage, making it one of the most abundant and widely distributed carbon feedstocks on Earth.^1^ Beyond its well-established role as a fuel, methane serves as a raw material for producing high-value chemicals including methanol, ammonia and hydrogen, underpinning a broad spectrum of industrial processes.^2^ Global natural gas reserves exceed 188 trillion cubic meters, representing a large untapped resource for chemical valorization.^3^ Despite this abundance, a substantial fraction of available methane is flared, vented or lost during extraction and transport, contributing to greenhouse emissions, where methane has a 20-year global warming potential approximately 80 times that of carbon dioxide (CO_2_).^4^ Therefore, the catalytic conversion of methane to methanol represents a strategically important and environmentally compelling transformation, enabling the upgrading of a volatile feedstock into a high-value chemical.^4^

The central challenge in methane-to-methanol conversion lies in the exceptional thermodynamic stability of the methane molecule. Direct methane-to-methanol conversion (DMTM) faces challenges due to the its C–H bond energy of 434 kJ mol^−1^, resulting in it being chemically inert.^5^ Therefore, strong oxidizing agents are necessary to facilitate C–H bond activation under moderate, non-hazardous operating conditions while preventing the overoxidation of the desired methanol product.^5^ Once methanol is formed, it is thermodynamically more reactive than methane, as its C–H bond energy is considerably lower (393 kJ mol^−1^).^6^ Traditional methods for converting methane to methanol, including high-temperature steam reforming of methane (SMR), are energy intensive, operating at temperatures of 800–1000 °C and high pressures up to 25 bar to produce synthesis gas (CO/H_2_) which is subsequently converted to methanol over a bimetallic catalyst, thereby minimizing the overall efficiency.^7^ This inefficiency has motivated the search for alternative methods that can convert methane to methanol under moderate and more sustainable conditions.

Catalytic DMTM conversion under relatively low-temperature and non-hazardous conditions has attracted significant research interest as a potentially transformative alternative to the conventional syngas route. A wide range of oxidants has been explored, varying in reactivity and practical applicability. Molecular oxygen (O_2_) is abundant and cost effective, but it requires precise control to prevent complete combustion to CO_2_. Hydrogen peroxide (H_2_O_2_) enables near-ambient, liquid-phase oxidation with high methanol selectivity, but it presents economic and safety challenges at scale. Nitrous oxide (N_2_O) can generate highly reactive atomic oxygen species, achieving exceptional methanol selectivity, although its classification as a greenhouse gas limits its industrial viability. Water (H_2_O) represents the most environmentally benign option, acting as a soft oxidant in stepwise cyclic processes.^8,9^

The development of catalysts capable of utilizing these oxidants with molecular-level precision, facilitating C–H bond activation while preserving the nascent C–O bond in methanol, remains a central challenge and defining frontier in this field. In these circumstances, metal–organic frameworks (MOFs) have attained attention as a promising set of catalysts for methane-to-methanol conversion. MOFs comprise metal clusters coordinated to organic ligands, contributing high surface areas, tunable structures, and well-defined pore networks that allow for selective interactions with methane molecules.^10^ Moreover, MOFs can act as both catalysts and precursors for MOF-derived materials like metal oxides and bimetallic composites, which strengthen their thermal and chemical robustness.^11^ Their scalability enables the tuning of metal nodes and organic linkers, which can aid in generating the active oxygen species required for methane activation, thereby improving methanol selectivity.

Hybrid systems involving MOFs and bimetallic nanoparticles (NP@MOFs) have revealed promising outcomes in overcoming challenges associated with methane activation and methanol selectivity. These methods benefit from synergistic effects that increase both catalytic activity and selectivity through tuning the oxidation states and electronic properties of the materials.^12^ Additionally, emerging techniques such as plasma-assisted, photothermal, and electrochemical activation are being investigated, opening new avenues for research and applications.^12^

This review provides a comprehensive overview of current advancements in methane-to-methanol catalysis, with a specific focus on MOF-based and MOF-derived catalysts. The discussion comprises catalyst design strategies, the role of different oxidants, reaction mechanisms, and structure–activity relationships. By inspecting these advancements, this review focuses on highlighting the potential of MOFs and their composites to overcome the challenges related to methane activation and methanol selectivity, thereby offering promising pathways for scalable and more efficient methane valorization.^13^

## 2 Catalytic oxidation of methane

Coherent methane-to-methanol conversion is crucial for reducing methane flaring and controlling its potential as a useful chemical.^8^ This process, however, is challenging due to the demands for green and practical methods for this approach.^9^ Catalysts and oxidants are the main keys to activating methane and achieving methanol production. The most common oxidants include O_2_, H_2_O, H_2_O_2_, and N_2_O, which are exceptionally effective due to their strong oxidizing properties and their ability to activate methane under mild conditions.^14^ Furthermore, activating O_2_*via* H_2_ or CO to generate H_2_O_2_ has been explored to promote methane oxidation efficiency and selectivity,^15^ while traditional solvothermal methods for synthesizing MOFs employ non-renewable solvents. Recent findings are shifting towards non-solvent-based, environmentally friendly approaches to upgrade sustainability and commercial feasibility.^16^

### 2.1 A new catalyst trend: MOF-based catalysts

MOFs have attained increasing focus for methane-to-methanol conversion due to their tunable porosity, high surface area, and structural flexibility.^17^ While zeolites, like Cu-ZSM-5 and Fe-MFI, and metal oxides, including Fe_2_O_3_ and TiO_2_, have shown high methanol selectivity and stability,^18^ MOFs are becoming promising alternatives. MOFs provide the potential for functionalization, such as the increase in bimetallic NP@MOF composites and MOF-derived metal oxides, which may solve scalability and stability challenges.^19^ While zeolites and metal oxides currently exhibit more practical benefits for commercial applications, the modularity and stability of MOFs set them up as a strong candidates for future advancements in methane oxidation.^20^

Catalysis plays a very important role in methane-to-methanol conversion, making it necessary to develop optimal catalysts for orderly and selective reactions. Metal nanoparticles (NPs) are consequential heterogeneous catalysts for methane oxidation, with unique surface modifications being crucial for improving performance.^21^ Incorporating metal nanoparticles into MOFs can refine catalytic efficiency, as MOFs offer well-defined and uniformly dispersed active sites that enhance selectivity and activity.^22^ MOFs, with their known high surface areas, tunable pore sizes, and structural flexibility, have distinct advantages over traditional catalysts, in particular metal oxides and zeolites.^23^ This review underscores how advances relating to MOF-based catalysts can manifest methane oxidation systems, illustrating connections with other catalytic areas for enhancing selectivity, activity, and stability.^9^

### 2.2 Nanoparticles in MOFs

Nanoparticles (1–100 nm) have attracted significant interest in the area of catalysis due to their high surface area, stability, and tunable physicochemical properties.^24^ Among the available options, metal and metal-oxide nanoparticles stand out for their catalytic activity in various oxidation processes, including methane-to-methanol conversion.^25^ Their small size enhances dispersion and access to active sites, allowing effective interactions with reactant molecules.^26^ Nanoparticles can be synthesized *via* physical, chemical, or green routes, each offering control over size, morphology, and surface functionality.^27^ When embedded within porous supports like MOFs, the resulting NP@MOF composites benefit from confinement effects, improved stability, and enhanced mass transfer.^14,28,29^ This synergy between nanoparticles and MOF matrices has been shown to promote selective C–H bond activation and suppress overoxidation, making NP@MOF systems promising platforms for mild and efficient methane oxidation.^16,30,31^ Researchers are exploring the possibility of combining MOFs with additional functional substances to create inventive composites with higher performance owing to unique characteristics.

Nanoparticle@MOFs offer a range of benefits, such as the ability of the nanopores to offer confinement effects and shape selectivity; exceptionally high porosity and surface areas, which are perfect for housing metal NPs; and the ability of suitable organic linkers in MOF structures to facilitate interactions with NPs.^32–34^ The vast variety and availability of MOF structures, with adaptable pore sizes and shapes, enables them to satisfy specific requirements for various metal NPs.^9,35,36^ Nanoparticle@MOFs have unique physical and chemical characteristics that make them excellent for use in a variety of disciplines, including physics, engineering, medicine, biology, chemistry, and materials science.^13,37–39^ Nanoparticles are considered an advanced technology owing to their rapid progress in several catalysis-related areas.^25^ The highly crystalline structures of MOFs containing nanoparticles make them promising materials for a range of applications, particularly in the preliminary treatment of samples for heterogeneous catalysis. This is attributed to their uniform open cavities, tunable pore sizes/shapes, permanent porosity, high surface area, and high thermostability.^29,40–43^

#### 2.2.1 Monometallic NP@MOF catalysts

The term “monometallic nanoparticles” (MNPs) refers to particles composed of a single metal, and their properties are determined by the metal atom that constitutes the particles. There are several types of MNPs, such as metallic, magnetic, and transition-metal nanoparticles, depending on the type of metal atom present.^32,44–47^ Monometallic nanoparticles are composed of a single metal element, which imparts its characteristics on the particles.^48–51^ Building monometallic atomic interfaces is a promising method because monometallic atomic interactions arise with shorter reaction routes and smaller interfacial barriers than standard multimetallic nanointerfaces, and the mass transport properties of catalysts may be modified.^52–55^ One method that shows promise for improving catalytic performance is the planned rebuilding of the initial MOF structure to achieve a desired structure, which can be assisted by analyzing the morphological changes of MOFs undergoing dynamic reconstruction during electrocatalysis, real-time product monitoring over the course of the reaction using *in situ* descriptors, and the determination of the actual active sites for reactions.^56^

#### 2.2.2 Bimetallic NP@MOF catalysts

Enhancing the catalytic efficiency of monometallic MOFs seems to be possible with the introduction of a second metal ion.^57^ Bimetallic MOFs are single-phase materials that react with organic linkers, instead of separate phases, and they involve a combination of various metal ions with almost the same electrical structure and density. Owing to the powerful interactions between nearby metal sites, bimetallic MOFs not only show excellent stability but also have the capacity to work in conjunction with each other to provide a possible synergistic effect that allows for the formation of tunable electronic structures and a greater variety of structures and attributes.^58^ More attention has been paid to bimetallic nanoparticles, which are made up of two distinct metals which, when combined, exhibit unique qualities unique to each metal. This is due to the fact that they are significant from both technological and industrial process viewpoints.^44^ Owing to the synergistic effects of the metals involved, bimetallic nanoparticles created by mixing various metals have the potential to introduce and enhance characteristics aimed at improving functionality.^59^ Bimetallic nanoparticles have attracted considerable attention due to their remarkable physical properties, which enable the integration of multiple metals to enhance optical and catalytic performance.^60^ Bimetallic MOFs have shown more impressive performance in gas adsorption compared with monometallic MOFs.^61^ In recognition of the distinctive properties of bimetallic materials, bimetallic MOFs also exhibit better catalytic performance than monometallic MOFs. In addition, bimetallic nodes added to the same MOF might end result in defects and amazing synergistic effects, increasing the number of active sites, thereby improving the catalytic activity even more.^62^ A brief comparison of the performances of different MOF catalytic systems specifically reported for methane-to-methanol conversion, highlighting reaction conditions, oxidants, and catalytic performance for direct comparison and the respective reaction products from methane-to-methanol conversion, is presented in Table 1.

**Table 1:** A comparison of the performances of different MOF catalysts and their reaction conditions

Catalyst used	Oxidant	Process	Conditions	Products	Yield/selectivity of methanol	Reference
Fe/Uio-66 (Fe-loaded MOF)	O_2_	Continuous flow reactor	Temp.: 180 °C	CH_3_OH	5.9 × 10^−2^ µmol g_cat_^−1^ h^−1^	63
Mil-53 (Al, Fe)	H_2_O_2_	Batch reactor	Temp.: 200 °C, time: 1 h, *P*: 30.5 bar	CH_3_OH, CH_3_OOH	20 µmol g_cat_^−1^ h^−1^, 80%	64
Mof-808 (Cu-loaded)	O_2_	Batch reactor	Temp.: 150 °C	CH_3_OH	Highly selective for methanol	65
Mil-100(Fe)@CdS	H_2_O_2_	Photo-batch	Ambient temp. + light	CH_3_OH	1000 µmol g_cat_^−1^ h^−1^	66
Mof-808-Iza-Cu	N_2_O	3 L custom-built adsorption vessel reactor	Temp.: 150 °C	CH_3_OH	61.8 µmol g_cat_^−1^ h^−1^, selectivity: 100%	67
MOF-808-His-Cu	N_2_O	3 L custom-built adsorption vessel reactor	Temp.: 150 °C, time: 1 h	CH_3_OH	31.7 µmol, selectivity: 100%	67
Cu_*x*_O_*y*_@UiO-bpy	O_2_	Fixed bed reactor	Temp.: 200 °C, time: 3 h	CH_3_OH and CH_3_CH_2_OH	24.33 mmol g_cat_^−1^ h^−1^, 88.1%	68
Cu/MoO3	O_2_	Fixed bed reactor	Temp.: 200 °C	CH_3_OH and CH_3_OOH	47.2 mmol g_cat_^−1^ h^−1^	69
Mof-808	N_2_O	Batch reactor	Temp.: 150 °C, time: 1 h, *P*: 1 bar	CH_3_OH	71.8 µmol g_cat_^−1^ h^−1^	66
Cu-Nu-1000	O_2_	Flow reactor	Temp.: 200 °C, time: 10 h, *P*: 40 bar	CH_3_OH	19.7 µmol g_cat_^−1^ h^−1^, 60%	70
Mof-808-Bzz-Cu	N_2_O	3 L custom-built adsorption vessel reactor	Temp.: 150 °C, time: 1 h	CH_3_OH	71.8 µmol g_cat_^−1^ h^−1^, selectivity: 100%	67

Table 1 showcases various MOF catalysts and related materials used for the direct conversion of methane to methanol (MTM), highlighting diverse catalytic strategies, reaction conditions, and performance metrics. One of the observed trends is the utilization of MOFs to stabilize active metal sites, such as copper (Cu) and iron (Fe), which are important for methane C–H bond activation.

The reported methanol yields and selectivities differ significantly across various catalyst systems and reaction conditions. For instance, the Fe/UiO-66 catalyst shows a methanol production rate of 5.9 × 10^−2^ µmol g^−1^ h^−1^ while operating in a continuous flow reactor at 180 °C.^63^ In contrast, MOF-808-Iza-Cu and MOF-808-His-Cu, both using N_2_O as the oxidant in a batch reactor at 150 °C, recorded higher productivities of 61.8 µmol g^−1^ h^−1^ and 31.7 µmol g^−1^ h^−1^, respectively, with 100% selectivity for methanol.^67^ Photocatalytic systems like MIL-100(Fe)@CdS offer an alternative approach, achieving a methanol yield of 1000 µmol g^−1^ h^−1^ at ambient temperature and under light irradiation with H_2_O_2_.^66^

The design strategies are diverse, aiming to enhance active site accessibility, stability, and selectivity. Many MOF-based catalysts, such as MOF-808 (Cu-loaded) and its derivatives (MOF-808-Iza-Cu and MOF-808-His-Cu), are designed to mimic the active sites of methane monooxygenase enzymes, often involving bioinspired Cu centers.^65,67^ Other strategies include the incorporation of single metal atoms or clusters within the MOF framework, as seen with Fe/UiO-66,^63^ or the use of polyoxometalates (POMs) immobilized within MOFs (POM@MOF) to create a protective and selective environment for methane oxidation.^66^ The choice of oxidant also plays a critical role, with N_2_O and H_2_O_2_ being commonly employed due to their ability to activate methane under milder conditions compared to O_2_.

Table 1 shows that MOF catalysts offer promising potential for methane oxidation under mild conditions, but ongoing improvements in catalyst design and the optimization of reaction conditions are needed to enhance catalytic efficiency for industrial-scale applications.

#### 2.2.3 Recent and efficient approaches for methane conversion to methanol

**Scheme 1:**
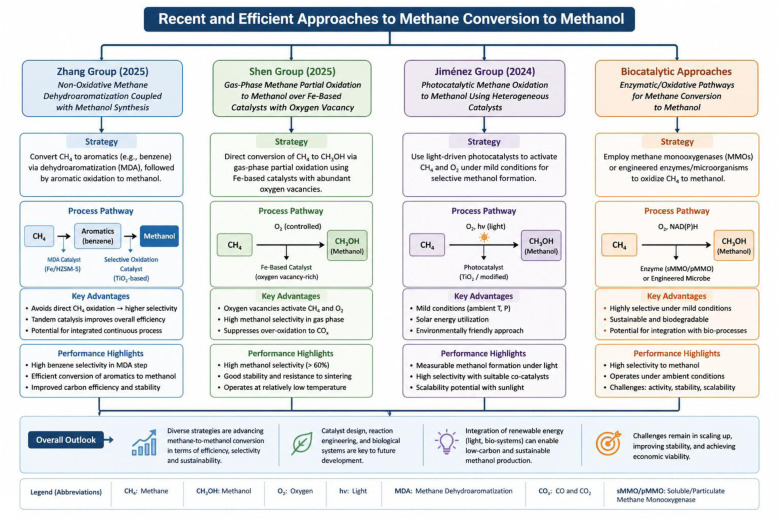
A flowchart summarizing representative strategies for methane-to-methanol conversion recently reported by research groups.

Methane-to-methanol conversion has been a subject of attention in recent years due to its potential for transforming an abundant and low-cost resource, methane, into a valuable chemical commodity, methanol (Scheme 1). The conventional industrial process for methanol production, the syngas route, is energy intensive and requires high temperatures and pressures.^71^ Consequently, research efforts have intensified to develop more direct, efficient, and environmentally benign methane-to-methanol conversion technologies, especially those operating under milder conditions.^72^ Recent breakthroughs in MTM conversion have focused on enhancing selectivity, improving reaction rates, and reducing the severity of operating conditions. These advancements include catalytic, catalyst-free, and biocatalytic domains.

An innovative catalyst-free approach reported by Zhang *et al.* in 2025 demonstrated the ambient conversion of methane to methanol using *in situ*-generated water dimer radical cations.^73^ This method achieved an incredible methanol yield exceeding 650 millimoles per hour and a conversion rate of approximately 35% under mild conditions. The high efficiency, reportedly 10^2^ to 10^6^ times higher than previously reported catalytic methods, is attributed to the unique redox properties of the water radical cations, which successfully activate the inert C–H bond in methane. The process also produces hydrogen gas as a valuable by-product. The absence of traditional catalysts and the mild operating conditions make this approach highly attractive for industrial scalability, overcoming issues related to the deactivation of catalysts and harsh reaction conditions.^73^

Catalytic approaches continue to evolve, with notable progress regarding designing highly active and selective catalysts. Shen *et al.*, in 2025, reported a highly efficient nickel-modified copper zeolite (Ni–Cu/ZSM-5) catalyst for the selective oxidation of methane to methanol. Using hydrogen peroxide (H_2_O_2_) as an oxidant in the liquid phase at 80 °C, a Cu_1_Ni_0.75_/ZSM-5 catalyst achieved a methanol yield of 82 162 mmol g_cat_^−1^ h^−1^ with selectivity of approximately 74%. The key to this enhanced performance lies in the promotional effect of nickel, which facilitates the conversion of Cu^II^ sites to more-active Cu^I^ sites. These Cu^I^ sites exhibit superior methane adsorption and activation capabilities, primarily due to a ligand effect that electronically perturbs the copper sites, making them more effective at cleaving C–H bonds.^74^ This approach illustrates the potential for high yields under relatively mild liquid-phase conditions.

Palladium (Pd)-based catalysts have shown considerable promise for direct methane-to-methanol conversion. Jiménez *et al.*, in 2024,^75^ developed monometallic Pd–CeO_2_ catalysts modified by interstitial carbon (Pd-iC–CeO_2_) through simple mechanochemical synthesis. These catalysts exhibited 100% selectivity towards methanol, a critical factor for industrial viability, as it minimizes unwanted by-products. Carbon modification plays a crucial role in preventing over-oxidation and stabilizing the active Pd sites on the ceria support. Another notable development involves PdAu/CeO_2_ alloy/oxide interfaces, which demonstrated remarkable selectivity (∼80%) at 500 K using water as an oxidant.^76^ The use of water as an oxidant is particularly attractive from an environmental and economic perspective. Furthermore, research into single-atom Pd in ZSM-5 catalysts has explored the role of oxidation-state-dependent reactivity in enhanced performance.^77^

Biocatalytic approaches offer an eco-friendly alternative, leveraging the power of microorganisms. Methanotrophic bacteria, such as *Methylosinus trichosporium* OB3b, naturally convert methane to methanol using methane monooxygenase (MMO) enzymes.^78^ Two main types of MMO exist: soluble MMO (sMMO), which has high activity and a broader substrate range, and particulate MMO (pMMO), which is more common in nature and exhibits higher affinity for methane.^79^

Recent advancements in this field include the metabolic engineering of methanotrophs to inhibit methanol dehydrogenase (MDH), an enzyme that typically further oxidizes methanol, thereby increasing methanol accumulation.^80^ Enzyme immobilization techniques are also being developed to improve the stability and reusability of MMO enzymes in industrial bioreactors. While offering ambient operating conditions and environmental benefits, biocatalytic conversion faces challenges related to gas–liquid mass transfer limitations (due to methane's low solubility in water), enzyme stability, and the cost of cofactors like NADH.^78^

The transition of these promising laboratory-scale technologies to industrial implementation requires addressing significant engineering and economic challenges. While direct methane-to-methanol conversion offers advantages over the conventional syngas route, several hurdles remain. As of 2025, the landscape of methanol production is witnessing a shift towards renewable and low-carbon sources. European Energy's Kassø e-methanol facility in Denmark, which commenced production in May 2025, exemplifies the move towards large-scale commercial plants, with a capacity of 50 000 tons per year. Projections indicate that global renewable methanol capacity could reach 7–14 million tons by 2030. Economic drivers of this transition include increased environmental regulations and carbon-pricing mechanisms. It was estimated that the levelized cost of methanol (LCOM) can range from $1283 to $1809 per ton, depending on the type of carbon capture and energy source.^81^ These economic incentives are driving more efficient and sustainable conversion methods.

The quest for efficient and industrially viable methane-to-methanol conversion continues to drive innovative research. Recent advancements in catalyst-free methods, advanced catalytic systems (*e.g.* Ni-promoted Cu/ZSM-5 and Pd-iC–CeO_2_), and biocatalytic routes offer promising pathways to overcome the limitations of conventional processes. The catalyst-free approach using water radical cations presents a paradigm shift with its high yields under ambient conditions, while advanced catalytic materials demonstrate exceptional selectivity and activity. Biocatalysis provides an environmentally benign route, though it faces challenges related to mass transfer and enzyme stability.

Focusing on catalytic conversion, continuous gas-phase catalytic oxidation using Cu-based catalysts, particularly Cu-zeolites, has emerged as the most recent and commercially promising technological approach out of other techniques. This process operates under relatively mild conditions of temperature and pressure (200–400 °C and 1–5 bar), which aligns well with industrial reactor designs and existing infrastructure. The use of simple oxidants such as oxygen (O_2_) or nitrous oxide (N_2_O) further strengthens its industrial viability, avoiding the need for costly and hazardous reagents like hydrogen peroxide. Moreover, Cu-based zeolite catalysts, which are used in industrial applications like NO_*x*_ reduction, have a vital advantage due to well-established industrial processes and their versatility. Therefore, while challenges affecting full industrial implementation exist, the progress recorded in both academic research and early-stage studies indicates strong potential for commercialization in the near future.

Industrial implementation will depend on addressing techno-economic challenges, particularly mass transfer, product separation, and oxidant cost. The increasing economic incentives from methane emission charges and the growing demand for renewable methanol are accelerating the development and deployment of these technologies. A phased implementation roadmap, as suggested for the broader methanol economy, will be crucial for integrating these diverse technologies into a sustainable and economically viable future for methane utilization. Continued interdisciplinary research and development, focusing on process intensification, novel reactor designs, and cost-effective oxidants, will be key to unlocking the full industrial potential of direct methane-to-methanol conversion.

Despite these promising advancements, significant challenges remain regarding industrial implementation. Key limitations include low methane conversion rates, high oxidant costs (particularly for H_2_O_2_), and difficulties in product separation.^82^ Among the various approaches, continuous gas-phase systems based on Cu-zeolites appear to be the most promising due to their compatibility with existing industrial infrastructure and their use of simple oxidants such as O_2_. However, further progress relating to catalyst stability, process intensification, and reactor design is required to achieve commercially viable methane-to-methanol conversion.

#### 2.2.4 Mechanistic pathways for methane-to-methanol conversion

**Scheme 2:**
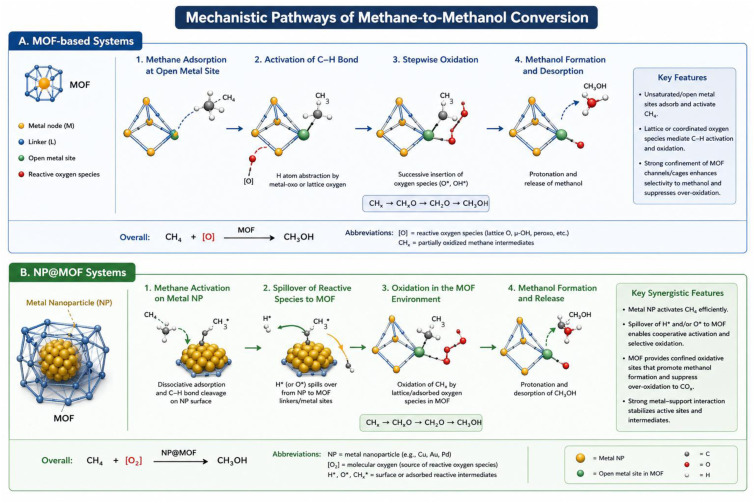
Mechanistic pathways for methane-to-methanol conversion over MOF-based and NP@MOF systems.

A mechanistic understanding of methane-to-methanol conversion over MOF-based catalysts is essential for the rational design of highly selective systems (Scheme 2).^66^ The intrinsic challenge arises from the need to activate the strong C–H bond in methane while suppressing the overoxidation of methanol, which is more reactive under oxidative conditions.^83^ MOF-based catalysts offer a unique platform to address this challenge due to their well-defined coordination environments, tunable metal nodes, and confinement effects, which enable precise control over reaction pathways at the molecular level.^84^ In MOF-based systems, methane activation generally occurs at isolated metal centers or open metal sites within the framework. These sites, often consisting of transition metals such as Fe, Cu, or Co,^85^ can generate reactive oxygen species upon interaction with oxidants (*e.g.*, H_2_O_2_, O_2_, or N_2_O). The mechanism typically follows a surface-mediated radical pathway, initiated by the formation of metal–oxo species (M=O), which act as the primary active sites for C–H bond activation. Hydrogen abstraction from methane produces a methyl radical (˙CH_3_) and a metal–hydroxyl intermediate (M–OH). The methyl radical is subsequently trapped within the confined pore environment and recombines with hydroxyl or oxygen species to form methanol.^86^

A defining feature of MOF-based catalysis is the site isolation effect, which prevents the aggregation of active metal centers and suppresses undesired side reactions. Unlike conventional metal oxides, where contiguous active sites may promote deep oxidation, MOFs spatially separate reactive centers, thereby limiting the radical chain reactions that lead to CO_2_ formation.^87^ This isolation mimics the behavior of enzymatic systems, such as methane monooxygenase, where controlled coordination environments enable selective oxidation. In addition, the confinement effect of MOF pores plays a critical role in modulating reaction intermediates.^88^ The porous architecture restricts the diffusion of methyl radicals and methanol molecules, increasing the probability of selective recombination while reducing exposure to further oxidation. This confinement also stabilizes transition states and lowers activation barriers for methane activation, contributing to enhanced catalytic efficiency under relatively mild conditions.^89^

For NP@MOF systems, where metal nanoparticles are encapsulated within the MOF matrix, the mechanism involves synergistic interplay between the nanoparticle surface and the surrounding framework.^90^ The nanoparticles facilitate the activation of oxidants and the generation of reactive O_2_ species, while the MOF shell regulates reactant diffusion and product desorption.^91^ This dual functionality enhances selectivity by combining high activity with a controlled reaction environment. Furthermore, in bimetallic MOF systems, cooperative interactions between different metal centers can tailor the electronic structure of active sites, improving both methane activation and methanol desorption while minimizing overoxidation.^92^

The choice of oxidant significantly influences the mechanistic pathway in MOF-based systems. H_2_O_2_ is widely used due to its ability to generate hydroxyl radicals or metal–oxo species under mild liquid-phase conditions, often resulting in high methanol selectivity.^93^ Molecular O_2_, although more practical for industrial applications, requires controlled activation to avoid complete combustion.^85^ N_2_O can produce highly reactive atomic oxygen species, but its use is limited by environmental considerations.^94^ In some cases, water participates as a soft oxidant or assists in methanol extraction from active sites, thereby improving selectivity.^95^

Despite these advantages, challenges remain in achieving continuous and scalable methane-to-methanol conversion using MOF-based catalysts. Issues such as framework stability under the chosen reaction conditions, limited methane solubility in liquid-phase systems, and oxidant utilization efficiency must be addressed. Future progress will depend on advanced active site engineering, defect modulation, and the development of robust MOF architectures capable of operating under industrially relevant conditions. Overall, MOF-based catalysts provide a highly promising platform for methane valorization, offering unprecedented control over catalytic mechanisms through structural tunability, site isolation, and confinement effects. These features position MOFs at the forefront of next-generation catalysts for selective methane oxidation.

#### 2.2.5 Metal–organic frameworks (MOFs) and bimetallic systems

**Scheme 3:**
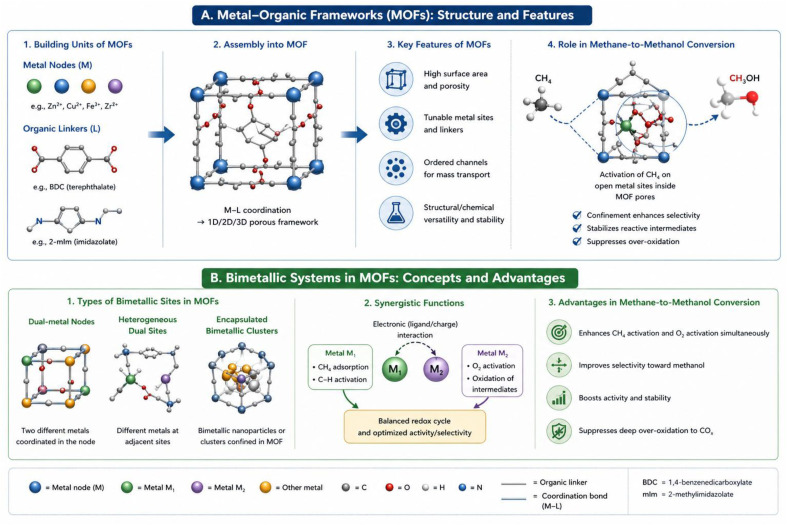
The structural features of metal–organic frameworks and synergistic effects in bimetallic systems.

MOFs are an emerging class of catalysts that offer incomparable tunability of active sites and pore environments (Scheme 3). MOFs like UiO-66 or ZIF-8 can enclose bimetallic nanoparticles (*e.g.*, AuPd or PdPt) or host isolated metal nodes that can mimic enzymatic function.^96^ The selectivity in MOF-based systems is usually attributed to the site-isolation and molecular screening properties of the framework, which can be modified to allow methane access while limiting the diffusion of overoxidation products.^96^

Bimetallic systems, like AuPd nanoparticles, improve selectivity by enabling the *in situ* generation of H_2_O_2_ or other reactive oxygen species under mild conditions. There can be a mutual effect between the two metals, for instance, Pd can provide the active sites for C–H activation and Au can moderate the electronic properties to prevent over-binding of methanol, which is very vital for maintaining high selectivity.^97^ In some cases, core–shell structures (*e.g.*, Pd@Pt) are employed to protect the active metal from sintering and to tune the surface reactivity toward partial oxidation.^83^

#### 2.2.6 MOF-derived catalysts

**Fig. 1:**
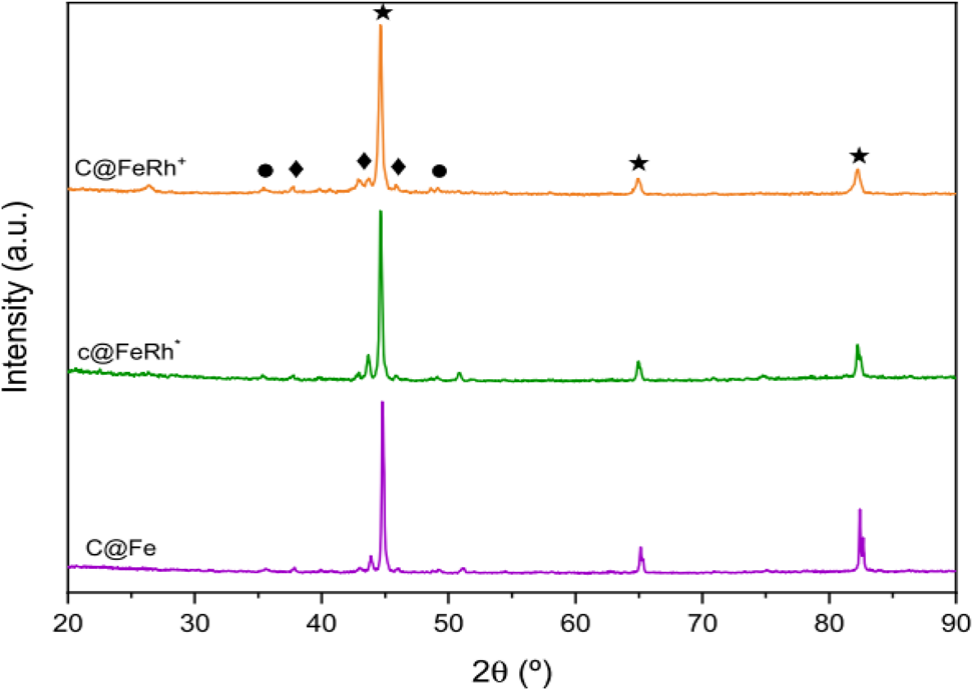
XRD patterns of as-synthesized catalysts (★ α-Fe, ◆ Fe_3_C, and ● Fe_3_O_4_).^110^

Considering their high catalytic activity and versatile tunability, MOF-derived materials are gaining popularity in the field of fine-chemical synthesis (Fig. 1). For example, MOF-derived catalysts have been used in coupling reactions, acid- and base-catalysis, and the oxidation and reduction of different functional groups, and they have been reported to have high selectivity and stability. The molecular composition and structural properties of MOF-derived supports, such as surface area and porosity, can be transformed to suit their intended applications.^98^ Materials produced or altered from an initial MOF structure, frequently to improve or modify certain attributes such as surface area, porosity, chemical stability, or functionality, are commonly referred to as derived MOFs. This derivation procedure could involve post-synthetic modification, which involves the incorporation of functional groups into the structure of a MOF, or additional chemical procedures to alter its characteristics after it has already been produced. Template synthesis involves creating new materials using a MOF as a blueprint or template. For example, calcining a MOF transforms it into a metal oxide or other carbon-based material. Developing composite materials with blended functions involves integrating MOFs with other materials, such as polymers or nanoparticles, and applications including medication delivery, gas storage, and catalysis, among others, are often studied using the derived MOFs.^99–101^ Owing to their uniform active sites, centralized porosity, compositional variation, ease of functionalization with other heteroatoms, and high stability, recently developed nanomaterials derived from MOFs have drawn more attention and demonstrated promising prospects for oxidation and hydrogenation catalysis.^102–105^ The excellent hydrogenation performance that exists at present for the selective hydrogenation of furfural, which is capable of achieving 100% selectivity and the complete conversion of furfural to furfuryl alcohol under 1.5 MPa H_2_ at 120 °C for three hours, is exclusively attributable to the catalyst's heterogeneity and excellent reusability.^106–109^ The MIL-100(Fe) MOF was pyrolyzed to create Fe-derived catalysts, which were subsequently examined for use in the reverse water–gas shift (RWGS) reaction. The addition of Rh as a dopant *via* wet impregnation or *via in situ* inclusion during synthesis was also considered. Furthermore, a C@Fe* catalyst exhibited the most effective performance at temperatures below 500 °C, which was attributed to the *in situ* incorporation of Rh during synthesis. Low Rh loading also results in a reduction in the particle size in the active phase (Table 2).^110–113^

**Table 2:** Textural properties of various catalysts^110^

Sample	*S*_BET_ (m^2^ g^−1^)	Pore volume (cm^3^ g^−1^)	Average pore size (Å)
C@Fe	51	0.10	77.11
C@FeRh*	59	0.13	85.31
C@FeRh	42	0.08	79.22

**Fig. 2:**
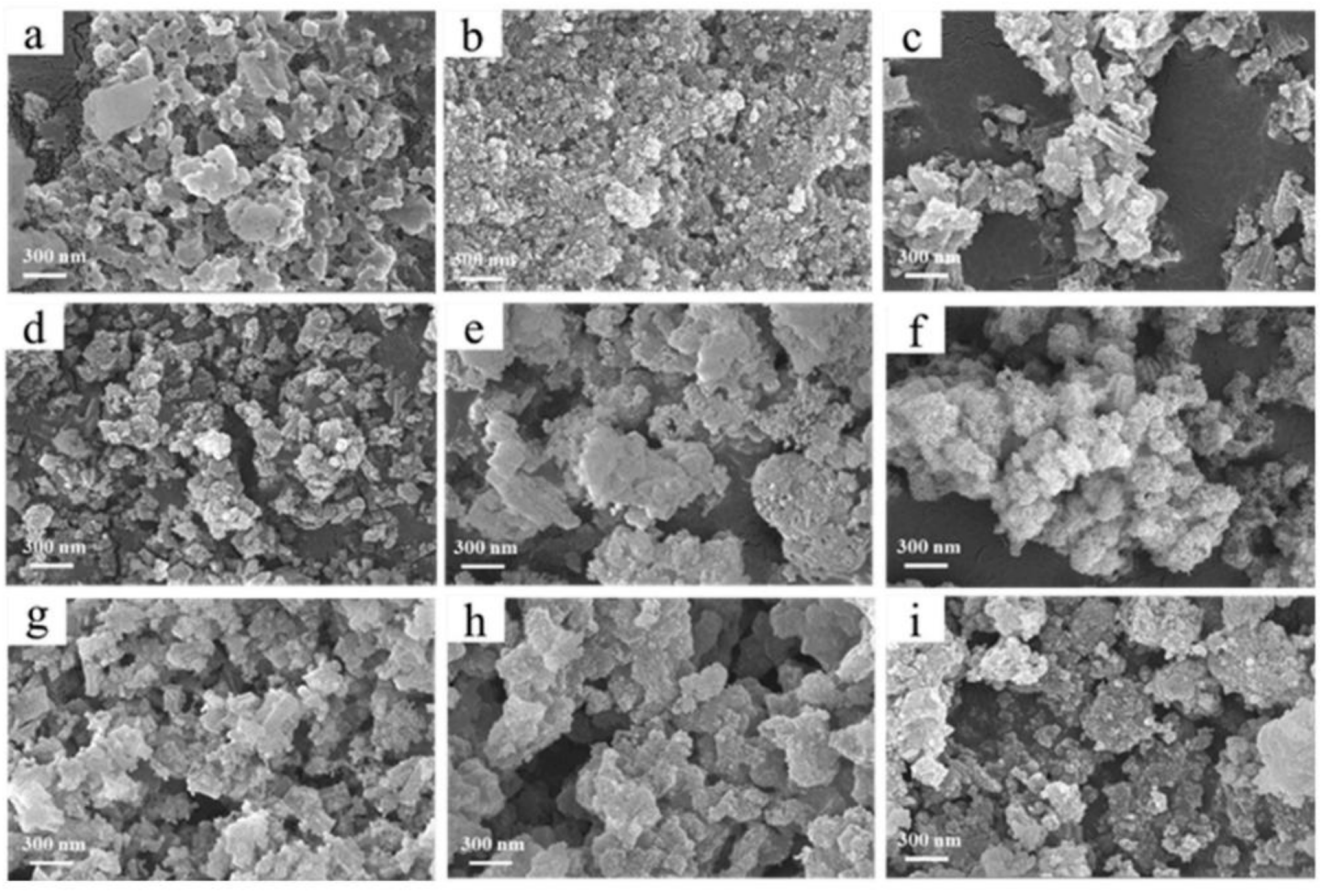
FESEM images of (a) Al-MOF, (b) Al_2_O_3_, (c) Ni–Al_2_O_3_-1%, (d) Ni–Al_2_O_3_-3%, (e) Ni–Al_2_O_3_-5%, (f) Ni–Al_2_O_3_-10%, (g) NiO–Al_2_O_3_-5%, (h) Ni–Al_2_O_3_-5%-used and (i) Ni-Al_2_O_3_-5 %-R.^128^

**Fig. 3:**
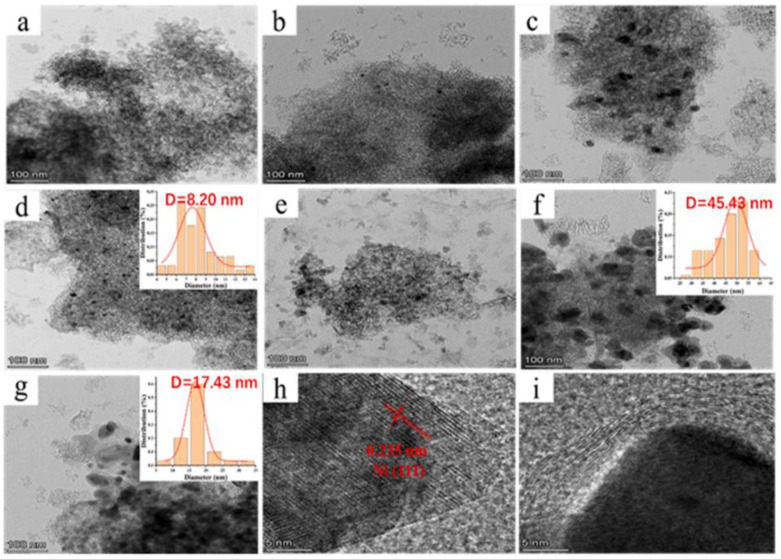
TEM images of (a) Ni–Al_2_O_3_-0%; (b) Ni–Al_2_O_3_-1%; (c) Ni–Al_2_O_3_-3%; (d) Ni–Al_2_O_3_-5%; (e) Ni–Al_2_O_3_-10%; (f) NiO–Al_2_O_3_-5%; (g) Ni–Al_2_O_3_-5%-used; (h) Ni–Al_2_O_3_-5%; and (i) Ni–Al_2_O_3_-5%-used. Insets: particle size distributions and diameters.^128^

As a result of the substantial binding energy between the oxygenated organic cluster and the active nanometal site, the high porosity of MOFs and stable coordinated bonds not only induce strong metal–support interactions (SMSIs) but also introduce a confinement effect because of their nanochannels (Fig. 2). Additionally, the high frequency of open unsaturated metal sites and active organic groups helps to enhance the catalytic sites of catalysts produced from MOFs.^58,114–118^ The impact of pyrolysis on MIL-101(Fe) was investigated and it was discovered that thermal treatment, including at 310 °C, revealed more Fe sites and oxygen vacancies (OVs) while retaining some of the original crystal structure.^119–121^ The catalyst Ni 40%–Ce-BTC, which had 40% nickel loading, showed improved qualities. These include decreased particle size, improved dispersion, improved reducibility, improved metal–support interactions, and reduced coke production. Consequently, following a 24-hour reaction on stream, its stability was examined at various reaction temperatures (500–800 °C), and it was discovered to be stable at those temperatures. The MOF structure confinement effect might be the source of the high stability of the catalyst, even at lower temperatures.^122–124^ Co-thermolysis of a Ni precursor and Al-MOF was used to create Ni–Al_2_O_3_ catalysts. The ability of the catalysts to pyrolyze PP catalytically into fuels was examined.^125,126^ The morphologies and characteristics of the catalysts were significantly affected by Ni loading. The distribution and composition of the products, as well as the effects of nickel loading, operating temperature, and species status, were investigated (Fig. 3). The catalyst can improve oil production at considerably lower temperatures and reduce undesirable wax in liquid products. Using Al-MOF-derived Al_2_O_3_, the maximum oil production (72.8%) from PP was achieved at 450 °C with 5% Ni loading.^127^ It was found that when more Ni was loaded into the catalyst, the pore size increased. The average observed pore size of Al_2_O_3_ without Ni loading was 10.7 nm. On the other hand, the catalyst showed its maximum average pore size of 19.0 nm when the Ni loading amount reached 3%.^52,128–130^

Table 3 highlights selected MOF-derived catalysts and their textural characteristics, which, while not entirely focused on methane oxidation, demonstrates the structural control and functional versatility of MOF-based systems.^47,148–150^ The ability to tailor surface area, pore volume, and active metal dispersion is highly relevant for designing catalysts capable of activating methane and stabilizing methanol intermediates. These properties are critical for suppressing overoxidation and enhancing methanol yield. The examples provided support the broader context of MOF-derived materials as a promising platform for engineering high-performance catalysts for methane-to-methanol conversion.^151–154^

**Table 3:** Textural properties of MOF-derived catalysts

Sample	Specific surface area (m^2^ g^−1^)	Pore volume (cm^3^ g^−1^)	Average pore size (nm)	Most frequent pore diameter (nm)	Reference
Zn-adipate MOF	63.6	0.039	2.46	—	131
Cu–BTC	1810	0.82	1.83	—	132
ZIF-67 (derived catalyst, inverse ZnO–Co structure)	972	0.45	2.3	1.7	133
Ag–Cu MOF (waste-derived, semiconductor functionalized)	187	0.196	2.05	—	134
Ni–LaAlO_3_ perovskite (MOF-gel precursor)	57.3	0.132	9.2	—	135
Co_3_O_4_/Ce_0_._6_La_0_._4_O_2_-*δ* (MOF-derived solid solution)	69.5	0.16	9.22	—	136
FeCu–MCOF (bifunctional MOF–COF hybrid)	224	0.214	3.8	—	137
CuO–ZnO clusters (from MOF precursor)	37.5	0.086	9.2	—	138
ZIF-8	1816	0.62	2.6	—	139
UiO-66	1120	0.44	2.1	—	139
MIL-53(Al)	1437	0.62	2.7	—	139
Ti-MOF-derived TiO_*x*_	112	0.12	4.3	—	140
Cu-MOF (for radical generation)	412	0.27	2.7	1.9	141
Mg-MOF-74	1138	0.47	2.3	—	142
3D cyclodextrin-MOF	653	0.38	2.3	—	143
Cu-PD-2-MBI MOF	328	0.21	2.6	—	144
ZIF-8 polymer coating	1835	0.63	2.6	—	145
MIL-53(Al) (pesticide sorption)	1456	0.59	2.4	—	146
Fe-BTC (pesticide sorption)	1215	0.52	2.5	—	146
Ag/Cu-modified ZIF-8	1556	0.61	2.3	—	147

IL-ZnCoFe@CNT/NC catalyst, a trimetallic catalyst with a distinct nanotube morphology grafted onto a 3D polyhedral carbon framework, exhibited exceptional catalytic effectiveness in the ORR with *E*_onset_ of 1.0 V, *E*_1/2_ of 0.89 V *vs.* RHE, and a low Tafel slope of 61 mV dec^−1^, underscoring its exceptional durability and efficiency.^127,155–157^ Carbon-supported catalysts (Co/C-n-*x* (*T*)) derived from lignin were produced using MOFs as templates and a straightforward hydrothermal process. Co/C-EL-0.5 (500) was the catalyst that supported the conversion of lignin-derived phenols the most. Its catalytic activity is significantly affected by its high specific surface area, excellent dispersion, small Co particle size, and appropriate acidity.^158–160^

SEM images and surface characterization results for various MOF-based and metal oxide catalysts demonstrate key structural features that directly influence catalytic performance. The particle size, as derived from SEM images, plays a vital role in determining the performance of a particular catalyst. The smaller the nanoparticles, the higher the surface area to volume ratio, which improves methane activation and the efficiency of methane-to-methanol conversion. The large surface area and porous structure of MOF-based catalysts expose more sites for the reactant to interact, which improves the reactivity and selectivity towards methanol and, at the same time, inhibits over-oxidation. The metal–support interaction (MSIs), which can be observed in SEM images, ensure the stability of the metal nanoparticles, which hinders sintering, thereby maintaining the catalytic performance. Structural flaws in the catalyst structure can be used to activate methane, but they must be monitored to avoid unwanted reactions, which might affect methanol selectivity. Therefore, the structural features observed from SEM imaging and surface characterization play a crucial role in improving the catalytic performance, which enhances the efficiency and stability of catalysts for the conversion of methane to methanol.

### 2.3 MOFs as catalysts for CH_4_ oxidation

**Fig. 4:**
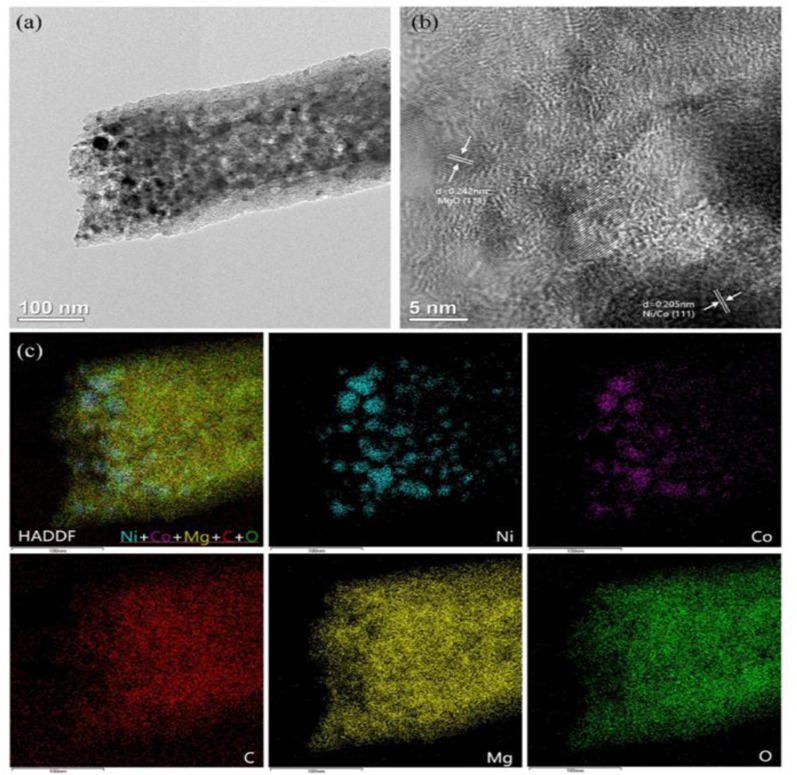
(a) TEM image, (b) HR-TEM image, (c) HAADF image and elemental mapping of the NiCoMg20 catalyst before the reaction.^168^

MOFs have several advantages when it comes to conversion. These include their vast surface area; porosity; recyclability, which allows for numerous cycles of recovery and reuse; and strong, adjustable structures and chemical characteristics,^161–163^ which allow for the insertion of accessible catalytic sites. Based on these distinct benefits, materials scientists have focused a great deal of interest on MOFs.^164^ MOFs are stable crystalline solids with a three-dimensional network structure, formed by coordinating organic ligands with metal ions. They have enormous surface areas, wide absorption ranges, and porous structures (Fig. 4).^165^ A great deal of research has been conducted to create more effective Ni-based catalysts to improve CH_4_ selectivity.^166^ MOFs have designable properties, and the excellent catalyst CQDs-25/NiMOFV achieved a highest yield and selectivity of CH_4_ of 6.0 mmol g^−1^ and 97.58%, respectively, which were approximately 3.22 times (1.86 mmol g^−1^) and 1.28 times (76.0%) higher than those of pure NiMOFV, respectively. A MOF-derived CeO_2_-supported Ni (Ni/CeO_2_-M) catalyst was easily produced under mild conditions, and its catalytic performance for DRM processes at 400–600 °C was investigated and contrasted with that of commercial CeO_2_-supported Ni (Ni/CeO_2_–C). CH_4_ conversion with Ni/CeO_2_–M at 550 °C was 30.8%, which was much greater than with commercially prepared Ni/CeO_2_–C (15.5%).^167^ Utilizing NiCoMg-MOF-74 as the precursor, MOF-derived NiCoMg@C catalysts were developed. This created a multi-metal system evenly dispersed within a carbon shell, producing catalysts with a high specific surface area and good dispersion. The dispersion of the active metals Ni and Co was further enhanced by the addition of a high content of magnesium, and when the molar ratio of Mg/Ni(Co) was 20, the greatest catalytic activity was obtained, where the initial conversion rate for CH_4_ was 75.13% (Table 4).^168^

**Table 4:** The porosity parameters of NiCoMg@C catalysts with various amounts of Mg^168^

Catalyst	BET surface area (m^2^ g^−1^)	BJH average pore volume (cm^3^ g^−1^)	BJH average pore diameter (nm)
NiCo@C	128.85	0.33	7.61
NiCoMg2@C	84.92	0.08	3.82
NiCoMg8@C	105.43	0.12	3.82
NiCoMg16@C	153.94	0.20	3.82
NiCoMg18@C	176.30	0.19	3.82
NiCoMg2o@C	231.67	0.22	3.82
NiCoMg22@C	320.48	0.24	3.42

### 2.4 MOFs for highly selective methanol production

The oxidation of methane to methanol using oxygen is a popular catalytic process; however, catalysts still have several difficulties relating to activity and selective oxidation, mostly in preventing over-oxidation. As a result, it is imperative to create new MOFs with enhanced catalytic efficiency and selectivity, as well as to investigate multi-metal nanomaterials, featuring bi-, tri-, and tetra-metals, for methanol oxidation.^169^ MOFs exhibit great potential as instruments for producing useful products with added value in several sectors. However, the oxidation of CH_4_ to MeOH using some current MOFs tends to be substantially weaker than that of fuels such as CO and CHOOH.^170^ MOFs provide a viable way to improve the energy supply while reducing greenhouse gas emissions and minimizing global warming. However, selectivity challenges regarding MeOH production need to be overcome.^171^ Improving product selectivity is a scientific challenge relating to the direct conversion of methane to methanol.^3,172–174^ Selectivity was analyzed in relation to the methanol concentration and the relationship between methanol oxidation and oxygen evolution.^125,175,176^ Under benign conditions, photocatalysis is an attractive pathway for the selective oxidation of CH_4_ to CH_3_OH. However, the selectivity and activity of CH_4_ oxidation are still far from satisfactory, owing to the ultra-high stability of CH_4_ and the wide range of oxidative products. To overcome these issues, dual-cocatalyst Au and bimetallic oxide (Zn_2_Ti_3_O_8_) nanoparticle (NP)-modified TiO_2_ nanotubes (Au/Zn_2_Ti_3_O_8_/TiO_2_) were explored for the highly selective and active photocatalytic oxidation of CH_4_ to CH_3_OH with O_2_ at room temperature. A produced amount of liquid oxygenates of 1200 µmol g_cat_^−1^ h^−1^ and CH_3_OH selectivity of 91.5% were attained by 1.7% Au/Zn_2_Ti_3_O_8_/TiO_2_-4 during photocatalytic CH_4_ oxidation, which were considerably higher than with 1.7% Au/TiO_2_ or 1.7% Au/ZnO.^177–180^ Improved performance in the catalytic oxidation of methanol was achieved with MOF-based materials, particularly in combination with different metals. Reducing the component size further increases the MOF activity. The addition of carbon-containing supports increases the catalytic capabilities by increasing the surface area and improving the dispersion of active materials.^181–184^ Exploring multi-metal nanomaterials, such as tri- and tetra-metals, may help advance methanol oxidation conversion *via* the combination of MOFs with multi-metal nanomaterials in a complementary fashion.^169^ Synthesized AuPd@ZIF-8 NPs for methane selective oxidation exhibited significantly improved performance over pure metals and monometal-doped ZIF-8, indicating that alloy metals and the ZIF-8 framework function in concert.^185,186^ In particular, production of liquid products up to 83.0 mmol g_AuPd_^−1^ h^−1^ and 89.5% selectivity were achieved at 50 °C with H_2_O_2_ (700 µmol) and 5 bar oxygen for 30 min.^187^ This review presents a wide range of catalysts, including transition-metal, noble-metal, and both monometallic and bimetallic systems, and it is important to clarify their catalytic reactivity and behavior during methane oxidation to methanol. A key challenge in this process is the activation of the strong C–H bond in methane (104 kcal mol^−1^), which requires the catalyst to generate highly reactive species (*e.g.*, lattice oxygen, surface-bound oxo species, or radical intermediates) without promoting overoxidation. Catalysts such as Cu-exchanged zeolites (*e.g.*, Cu–ZSM-5) and Fe-based MOFs have shown promising activity by enabling C–H activation through isolated metal sites or redox-active centers. However, one of the critical limitations across many systems is the overoxidation of methanol to unwanted byproducts, which is thermodynamically favored under oxidative conditions. Effective catalysts typically operate under stepwise or mild conditions, using oxidants like N_2_O or H_2_O_2_ to limit excessive oxidation. Future research should prioritize understanding the structure–activity relationships of these catalysts, especially how active site design, oxidation state, and reaction environment influence both methane activation and methanol selectivity.

### 2.5 Techniques used for CH_4_ oxidation to produce methanol

Methane-to-methanol conversion has been investigated using various catalyst systems and techniques. Composite catalysts like Fe and Co oxides supported on HZSM-5 have shown promising activity in fixed-bed microreactors, and they have been evaluated using methods such as H_2_-TPR, XPS, XRD, FTIR, and UV-vis spectroscopy to understand product distribution and selectivity.^188^ Photocatalysts like vanadium-doped mesoporous silica enable methane activation under ambient conditions by generating photoactive triplet states and suppressing overoxidation.^189^ Transition metal (*e.g.* Cu, Fe, and Co)-exchanged zeolites operate at 210–300 °C using oxidants like N_2_O, O_2_, or H_2_O_2_, with their performance enhanced by added mesoporosity and water vapor.^190^ CuO clusters and water vapor are used in chemical looping strategies for selective methane oxidation.^191^ Cu/MoO_3_ catalysts prepared *via* impregnation have achieved high STY CH_3_OH values, with characterization confirming CuMoO_4_ formation as the active phase.^69^ Cu-exchanged mordenite zeolites function *via* stepwise steam extraction and methane loading, with active Cu species identified through FT-IR, HERFD-XANES, and XPS.^192^ Electrochemical strategies use catalysts that generate active oxygen species to selectively oxidize methane while preventing methanol overoxidation.^193^ Recent materials like graphene and MOFs when combined with nanomaterials have shown improved methanol selectivity under mild conditions. These methods either directly activate methane or follow a two-step approach *via* syngas.^66^ Solid-state ion exchange introduces Cu into zeolites to form Brønsted and Cu-oxo sites, which selectively activate methane after thermal treatment.^194^ Reactors like fixed-bed, batch, and plasma reactors (microwave, DBD) are employed to overcome energy barriers and facilitate the formation of intermediates such as 
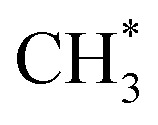
.^12,14,195,196^ Ion exchange techniques such as SCIE and AIE, along with *in situ* FTIR and H_2_-TPR, help identify active copper species for improved methanol yields.^197^ Photocatalytic oxidation using Pt-modified TiO_2_ under light enhances selectivity by generating hydroxyl and methyl radicals.^198^ Copper ion-exchanged zeolites such as those with mono (µ-O), dicopper or [CuOH]^+^ species serve as principal catalysts due to their ability to activate methane's C–H bond, with water acting as both extractant and oxidant.^199^

### 2.6 Emerging catalytic approaches: photocatalytic and electrochemical methods

In the last several years, photocatalytic and electrochemical methods have been more extensively investigated as promising avenues toward methane-to-methanol conversion under mild conditions. Photocatalysts such as TiO_2_-based materials with dopant or noble-metal modifications have been shown to have enhanced light absorption and selectivity. Researchers^200^ prepared a Pt/TiO_2_ photocatalyst with more than 72% methanol selectivity upon visible light irradiation. It was also^201^ discovered that Cu doping of graphitic carbon nitride improved room-temperature photocatalytic CH_4_ activation. On the electrochemical front, Fe–Ni-based electrocatalysts supported on nitrogen-doped carbon achieved high faradaic efficiency (∼48%) in relation to methanol under ambient conditions.^202^ Other critical systems include single-atom catalysts anchored on conductive supports and dual-function electrodes combining CH_4_ activation and *in situ* oxidation pathways.^203^ Hybrid photoelectrochemical techniques are also being explored, combining photocatalysis and electrochemistry in single setups to achieve solar and electrical energy synergy.^204^ Although advancements have been encouraging, selectivity, long-term stability, and scalability concerns remain. Active-site engineering, light harvesting, and electrolyte optimization are areas in which research is key to fill the gap between industrial applications and laboratory discoveries.

## 3 Conventional catalysts: supported catalysts for methane oxidation

Cu, Ni, Zn, Fe, and Co are among the transition-metal catalysts that have been supported by lignin-derived charcoal (BC). During the selective oxidation of methane to methanol (SOMM) process, Cu/BC showed the greatest methanol output, obtaining 686.92 µmol g_Cu_^−1^ h^−1^ under ideal conditions and retaining catalytic activity for 18 h. Transition metals increase the carbonization of BC, facilitating methane adsorption and the dispersion of active species.^16,205–207^ HZSM-5-supported Fe–Co oxide catalysts were used for the direct conversion of methane to methanol. Different comparable iron and cobalt oxide compounds were found using characterization methods, and they were affected by the total metal oxide loading and Fe/Co molar ratio. During the reaction process, the catalysts showed a synergistic effect that improved the methanol yield and selectivity, especially at an equimolar ratio of iron to cobalt.^188,208^ Mn, Fe, Co, Ni, and Cu are all transition-metal catalysts that have been supported by vitreous silica for direct non-oxidative methane coupling (NMC). The M/SiO_2_-v catalysts were produced by fusing quartz and metal silicate precursors in a flame. Among them, Co performed the best, balancing hydrocarbon output, a low coke yield, and methane activation, whereas Fe and Co displayed greater hydrocarbon selectivity than the other metals.^209^ A hierarchical ZSM-5 zeolite, which was produced with intra- and inter-crystalline mesopores, supported cobalt oxide (Co_3_O_4_). The physicochemical features of the catalysts differ. Co-oxide/ZSM-5 with inter-crystalline mesopores has better catalytic activity for methane partial oxidation, mainly producing formaldehyde, whereas the intra-crystalline variations primarily generate methanol.^210^ Zinc titanate (ZnTiO_3_)-supported silver (Ag) was produced using an impregnation technique. Transmission electron microscopy and X-ray diffraction were employed to evaluate the catalysts, and the results confirmed that metallic Ag silver nanoparticles were uniformly dispersed over the ZnTiO_2_ surface. The catalytic activity of Ag nanoparticles in the photocatalytic oxidation of methane to methanol is influenced by their particle size, which is 10 nm at 5 wt%.^211^ Beta zeolite modified with cerium oxide (CeO_2_) and zirconium oxide (ZrO_2_) was used to support palladium (Pd) catalysts. Strong hydrothermal stability and good methane oxidation performance were demonstrated by the Pd/6.8Zr-beta catalyst, which reached T50 and T90 values of 417 °C and 451 °C, respectively. In contrast, CeO_2_ aggregation lowered the efficiency of the Pd/8.6 Ce-beta catalyst and produced inactive Pd^4+^ species, highlighting the importance of support selection for catalytic activity.^212^ For the oxidative coupling of methane (OCM), MnO_*x*_–Na_2_WO_4_ catalyst supported by silicalite-1 (S-1) shows high catalytic activity. The uniform distribution of active components on the S-1 support resulted in more active sites and increased stability. The weakly bound lattice oxygen in this catalyst facilitates the production of electrophilic species, which is essential for attaining a high C_2_ yield of 24.2% at 760 °C.^213^ Palladium (Pd) catalysts were supported by zeolites for the oxidation of methane, and the benefits of this support type over others, such as alumina, should be noted, including its superior resistance to water inhibition. This illustrates the key function of silanol nest defect sites in enhancing the stability of finely dispersed Pd nanoparticles, which is essential for efficient methane oxidation at low temperatures. It also highlights the significant role of the Si/Al ratio in determining the catalytic activity and stability, with higher ratios leading to improved performance.^214^ A colloid-impregnation approach was used to produce supported iridium (Ir) catalysts, including Ir/SiO_2_. At low temperatures, these catalysts displayed remarkable activity in lean methane oxidation, particularly when the Ir particle size was less than 2.0 nm and mostly formed of Ir^3+^ species. Particle agglomeration decreases the catalytic effectiveness at higher calcination temperatures. Supports that fortify the metal–support contacts can lead to enhanced thermal stability, suggesting their potential relevance.^215^ Pd–Ni bimetallic catalysts based on CeO_2_ were synthesized by ball milling (BM) and incipient wetness impregnation (IWI). The catalysts demonstrated improved performance in the partial oxidation of methane (POM), with the highest methane conversion and syngas output observed at optimum compositions of 0.12 wt% Pd and 1.38 wt% Ni. These materials are important for catalytic activity, as they show excellent stability and high metal species dispersion on the CeO_2_ surface.^216^ Ni nanoparticles were supported on γ-Al_2_O_3_ modified by Fe, with Fe ions evenly distributed within the support matrix. When the partial oxidation of methane is carried out, an ideal Fe mole percentage of 0.03 greatly improves coking resistance and inhibits the production of unwanted phases such as FeAl_3_O_4_ and NiFe alloys. The stability and effectiveness of the catalyst during the reaction are enhanced by the presence of Fe^3+^ ions, which facilitate carbon oxidation and removal.^217^ Pd catalysts, which are essential for the efficient oxidation of methane, were supported by metal oxides. Strong metal–support interactions, particularly with reducible metal oxides such as CeO_2_ and Co_3_O_4_, improve the efficiency of these catalysts. Usually, a mixed state of PdO and Pd(0) constitutes the active phase, with particle sizes ideally ranging between 5 and 10 nm. The catalytic activity is greatly influenced by the existence of oxygen vacancies and the capacity to transport reactive oxygen species.^218^ Palladium (Pd) catalysts supported on foam ceramics by a freeze-dried Ce–P/γ-AlO_3_ coating consequently increased the specific surface area and formed mesoporous structures that promoted the interaction of cerium (Ce) and phosphoric acid (P) with Pd. By successfully preventing nanoparticle aggregation, the freeze-drying method increases the number of active sites and optimizes the CO oxidation and CO methanation reaction performance.^14,219^

### 3.1 Monometallic catalysts

**Fig. 5:**
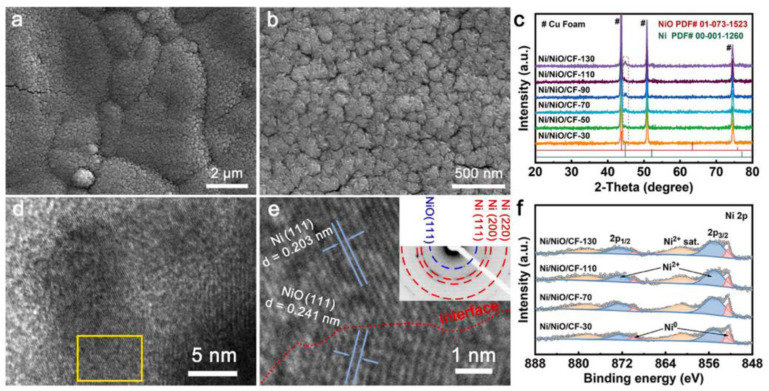
The physical characterization of Ni/NiO/CF-*x* catalysts: (a and b) SEM images of Ni/NiO/CF-110, (c) XRD patterns, (d) HRTEM image of Ni/NiO/CF-110, (e) an enlarged view of the highlighted region in (d) and an SAED image of Ni/NiO/CF, and (f) XPS analysis.^223^

The catalytic activities of palladium (Pd) and platinum (Pt) monometallic catalysts have been studied. Pt and Pd were used in the competitive oxidation process of methanol and ethanol specifically using alumina, SBA-15, faujasite-type Y (FAU-Y), and ZSM-5 zeolites as catalysts. It was demonstrated that the Pt/FAU-Y catalyst performed the best because of its optimized active sites, which are more accessible to methanol molecules than ethanol, regardless of the support's pore shape and variations in molecular size.^220^ The monometallic iron catalyst Fe@ZrO_2–10_ is composed of 10% zirconia with iron as the active metal. Having a space-time yield (STY) of 250 mg_HA_ h^−1^ g_cat_^−1^ and a selectivity to higher alcohols (SHA) of 30% under ideal circumstances, this catalyst outperforms other documented monometallic Fe-based catalysts in the production of higher alcohols.^221^ Nickel (Ni), copper (Cu), iron (Fe), cobalt (Co), platinum (Pt), palladium (Pd), iron (Fe), and rhenium (Re) are monometallic catalysts that have been examined. The noble metals were only used at 0.5% due to solubility constraints, whereas these catalysts were supported on silica at concentrations of 4% and 0.5% for non-noble metals. These metals were chosen because of their ability to selectively deoxygenate and their possible performance in the catalytic processes under investigation.^222^ A monometallic interphase synergistic catalyst, or Ni/NiO composite, was made of nickel and nickel oxide. In the hydrogen evolution process (HER), this catalyst, which is self-supported on copper foam, performs admirably, yielding a low overpotential of 22 mV at 10 mA cm^−2^ and strong stability over 100 h at significant current densities (Fig. 5).^223^

Ni/γ-Al_2_O_3_, or nickel supported on γ-Al_2_O_3_, was used as a monometallic catalyst. Compared to bimetallic catalysts, this catalyst was more effective in reducing the oxygen content of bio-oil produced from soybean meal; it was indicated that the Ni/γ-Al_2_O_3_ catalyst performed better in the hydrodeoxygenation process by producing bio-oil with the highest carbon and hydrogen content and the lowest oxygen content.^224^ The application of cobalt (Co) and molybdenum (Mo), two monometallic catalysts, was studied. These catalysts were evaluated for their efficiency in catalytically breaking down light cycle oil (LCO) to create monoaromatics such as benzene, toluene, and xylene (BTX). They are supported on zeolite materials, notably beta and Y zeolites.^225^ Iron (Fe) and nickel (Ni) monometallic catalysts were used to catalyze the breaking of polypropylene to produce hydrogen. Upon evaluation of their performance, the nickel catalyst produced a gas yield of 214.28 mmol g_PP_^−1^ and a hydrogen yield of 134.89 mmol g_PP_^−1^, whereas the iron catalyst produced gas at a rate of 172.09 mmol g_PP_^−1^ and hydrogen at a rate of 112.71 mmol g_PP_^−1^.^226^ The synthesis and evaluation of SnO_2_ catalysts doped with monometallic elements was carried out, with an emphasis on doping with Al and Sb. In density functional theory (DFT) calculations, which aided in the experimental validation of the catalysts for the selective catalytic oxidation of ammonia (NH_3_–SCO), these elements were chosen according to their positions with respect to the O p-band center.^227^ Biodiesel was produced by transesterification using monometallic oxide catalysts, namely calcium oxide (CaO) and cerium oxide (CeO_2_). To assess the catalytic stability and effectiveness of these catalysts, they were compared with the newly developed gCaO–CeO_2_ bimetallic oxide catalyst. This study emphasizes the shortcomings of monometallic catalysts, namely, their limited porosity and instability.^228^ An inexpensive copper monometallic catalyst supported on a MOF based on chromium, 3% Cu@MIL-101(Cr), was studied. To efficiently produce aromatic monomers, this catalyst was designed for the hydrogenolysis of lignin in supercritical ethanol. Employing this copper catalyst in an inventive manner provides a competitive substitute for the noble-metal catalysts that are typically utilized in comparable procedures.^229^ Several monometallic catalysts, such as ruthenium (Ru), palladium (Pt), nickel (Ni), molybdenum (Mo), and platinum (Pt), have been studied. After synthesis, the effectiveness of these catalysts in producing hydrocarbons from vegetable oil feedstocks with high free fatty acid concentrations was assessed. During hydrotreatment, the Ni and Mo catalysts demonstrated noteworthy performance and selectivity in the production of linear saturated hydrocarbons.^230^ Cobalt (Co) and iron (Fe) monometallic catalysts are supported by alumina. The iron and cobalt catalysts were thermally treated at 500 °C and 700 °C, respectively, under nitrogen flow. Prior to activity testing, both types of catalysts were reduced, and their effectiveness was assessed in the ammonia breakdown process to create hydrogen without CO_*x*_.^231^ Nickel (Ni), copper (Cu), and palladium (Pd) are three monometallic catalysts that have been used. Before reduction, the average diameters of the particles in these catalysts were measured and reported to be 4, 5, and 9 nm for Pd, Cu, and Ni, respectively. The diameters of Pd, Cu, and Ni became 8, 35, and 10 nm, respectively, after reduction, showing that Cu was more likely than the other metals to sinter under reducing conditions, as demonstrated in Table 5.^232^

**Table 5:** The textural properties of monometallic and bimetallic catalyst samples^232^

Sample	Nominal molar ratio (Pd : Cu@Ni)	Metal loading^a^ wt%				Surface area^b^ (m^2^ g^−1^)	Pore diameter^c^ (nm)	Crystallite size^d^ (nm)		TEM particle size^e^ (nm)
		Pd	Cu	Ni	Total			Pre-reduction	Post-reduction	
Norit carbon	—	—	—	—	—	660	4.0	—	—	—
Pd	1.00	4.8	—	—	4.8	535	3.8	4.0	8.0	8.0
Cu	0.01	—	3.6	—	3.6	547	3.8	5.0	35	17.3
Ni	0 : 01	—	—	4.9	4.9	562	3.9	9.0	10	8.5
Pd6oCuao	3.02	3.1	1.2	—	4.4	596	3.9	—	—	8.1
Pd4oCuee	2.03	2.5	2.2	—	4.6	450	3.8	—	—	10.6
PdzoCuse	1.04	1.3	3.3	—	4.5	580	3.8	—	—	8.9
Pd6oNi40	3.02	3.3	—	1.2	4.5	575	3.9	—	—	8.0
Pd.coNioo	2.03	2.3	—	2.0	4.3	572	3.9	—	—	7.7
Pd2oNino	1.04	1.4		2.9	4.3	517	3.8	—	—	7.6

a ICP-OES.

b BET.

c BJH.

d XRD. The reduction of the catalysts was conducted at 450 °C for 2 h.

e TEM.

Propane dehydrogenation was carried out using monometallic platinum (Pt) catalysts based on γ-Al_2_O_3_. In particular, ultradispersed Pt catalysts on bare and potassium-modified alumina (Al_2_O_3_-0.5 K) were investigated at loadings of 0.1, 0.2, and 0.3 wt%. The results show that, although similar turnover frequencies are retained across various Pt loadings, the addition of potassium dramatically affects the catalytic performance, especially in terms of stability and deactivation due to coke deposition.^233^ A mononuclear Rh(i) complex was used as a precursor for monometallic catalysis. The complex was produced *via* the reaction of N,N-donor ligands with [Rh(COD)(MeCN)_2_]BF_4_, and it was identified by its water solubility. When hydroformylating 1-octene, it exhibited strong activity, achieving notable conversions and excellent aldehyde chemoselectivity without generating alcohols or alkanes as byproducts of hydrogenation. However, in the same procedures, it exhibits less activity than its binuclear equivalent.^234^

#### 3.1.1 Noble metals

Noble metals, such as palladium (Pd), platinum (Pt), iridium (Ir), rhodium (Rh), ruthenium (Ru), and mercury (Hg), have been used to convert methane to methanol. However, the practical application of these noble metal complexes is hampered by issues such as low turnover rates, high prices, difficulties in separation, and reactor corrosion.^235^ Ruthenium (Ru), a noble metal, is a catalyst that transforms methane into methanol. Under moderate conditions, Ru single-atom catalysts (SACs) anchored on sulfated zirconia (SZ) efficiently activate methane (CH_4_) to produce methanol (CH_3_OH). The high acidity of Ru/SZ SACs promotes the breakdown of hydrogen peroxide (H_2_O_2_) into hydroxyl radicals (˙OH), which increases the overall catalytic activity and methanol production.^236^ Methane was converted to methanol using gold (Au). Au nanoparticles (NPs) were introduced to the photocatalyst to promote charge separation, which improved methane (CH_4_) and oxygen (O_2_) adsorption and activation. This substantially boosts the photocatalytic activity. A high yield of methanol (CH_3_OH) with approximately 100% selectivity was achieved as a result of this synergistic operation.^21^ Noble metals with near-100% selectivity and stability over extended periods of time, such as Ru, Pd, and Rh, are very useful in the conversion of methane to methanol. However, their high cost prevents them from being widely used in the CO_2_ methanation process.^237^ One noble metal that is significant for the process of transforming methane into methanol is ruthenium (Ru). With its excellent stability, catalytic activity, and low carbon deposition, it is a suitable catalyst for dry reforming methane (DRM) processes. Palladium (Pd) and platinum (Pt), on the other hand, exhibit deactivation as a result of sintering and carbon production.^238^ Ru, Au, Pt, and Pd are noble metals utilized in the process of converting methane into methanol. Ru had a considerable effect on the methanol yield, increasing it to 157.36 µmol g_cat_^−1^ h^−1^, whereas unaltered Cu-ex-MOR only produced 12.89 µmol g_cat_^−1^ h^−1^. While Pd slightly decreased the yield, the addition of Au and Pt increased the yield. Ru performs better than the other materials because of its great dispersion, enhanced surface acidity, and increased oxidation sites, which assist in activating methane.^239^ Rhodium (Rh) is a noble metal used to convert methane to methanol. Rh-based catalysts are highly resistant to the production of carbon during the dry reforming of methane (DRM), which makes indirect dissociation mechanisms an efficient way to activate methane. Consequently, the catalyst surface experiences less carbon accumulation, improving its stability and reaction performance.^240^ Palladium (Pd) is frequently used to convert methane to methanol. It has good catalytic activity and is more stable and less vulnerable to sintering in high-temperature reactions when coated with porous materials, such as CeO_2_. As a result, methane was burned efficiently, and the overall catalytic performance was enhanced.^241^ Noble metals such as platinum (Pt), palladium (Pd), ruthenium (Ru), and rhodium (Rh) are commonly used in the process of converting methane into methanol. In methane reforming processes, these metals improve the stability, selectivity, and activity. However, they are not viable for large-scale industrial applications because of their high cost and restricted availability.^242^ Rhodium (Rh) is a noble metal that is utilized to convert methane to methanol. Catalysts based on Rh, include 0.1% Rh/Al_2_O_3_.^243^

#### 3.1.2 Transition metals

The transition metals nickel (Ni), cobalt (Co), and iron (Fe) are used to convert methane into methanol. By aiding the dissociation of methane molecules, these metals improve the catalytic activity and efficiency. The atomic ratio of the metal affects the effectiveness of the product, with mixed oxides performing optimally.^244^ The two main transition metals utilized in methane-to-methanol conversion processes, such as bi-reforming of methane (BRM), are nickel (Ni) and cobalt (Co). Given its high activity for methane dissociation, nickel can be especially effective, whereas cobalt enhances catalyst stability by being less likely to sinter. However, problems such as carbon deposition can affect both metals and reduce their catalytic potency.^245^ The metals nickel (Ni), rhodium (Rh), and palladium (Pd) have been used to convert methane into methanol. With no signs of deactivation for a time on stream (TOS) of 96 h and the highest turnover frequencies (TOFs) for methane and carbon dioxide conversion, monometallic Rh-based catalysts stood out in terms of activity and stability, suggesting their efficacy in the dry reforming of methane.^246^ The process of converting methane to methanol using non-precious transition-metal catalysts has been studied, specifically nickel (Ni) and its alloys with other metals such as iron (Fe), tin (Sn), and manganese (Mn). The introduction of Sn into Ni can weaken carbon binding and enhance methane activation, leading to better catalytic performance. These catalysts improve carbon tolerance by changing the binding energies of carbon species, which helps reduce carbon deposition and catalyst deactivation during the dry reforming of methane (DRM).^247^ Molybdenum nitride (Mo_2_N) is a transition-metal-based catalyst used in the conversion of methane to methanol. Furthermore, Mo–N supports have been successful in promoting the dispersion of active sites and improving the total catalytic performance in methanol synthesis.^248^ The transition metal nickel (Ni) is used to convert methane to methanol, and palladium (Pd) is frequently used in conjunction with Ni. Pd promotes the reduction and dispersion of Ni, resulting in an increase in the number of active sites that speed up the methanation process. By facilitating the binding of formyl ligands and enhancing hydrogen adsorption, a mechanism essential to the conversion process, this combination facilitates the effective synthesis of CH_4_.^249^ Copper (Cu) is a widely recognized transition metal that is utilized to convert methane to methanol and has shown remarkable efficiency in this process. Its electrical characteristics improve the reaction kinetics and selectivity for methanol synthesis by facilitating the activation of methane. Furthermore, the number of active sites on the catalyst support is greatly increased by the dispersion of copper, which enhances reactant adsorption, surface interactions, and critical processes for increasing catalytic activity. While other metals, such as iron (Fe) and nickel (Ni), have also been investigated, the superior performance of copper-based catalysts has attracted the greatest interest.^195^ Cu-loaded MOFs containing the copper (Cu) metal required to convert methane to methanol have demonstrated notable improvements in catalytic performance, particularly when paired with other metals such as Zn. This mixture improves the catalytic activity and selectivity, which increases the methanol yield and accelerates reactions.^250^ Nickel (Ni) is utilized to convert methane to methanol. Yttria-stabilized zirconia (YSZ)-supported nickel-based catalysts have demonstrated high methane production rates and selectivity, which are made possible by incorporating nickel, which also reduces problems such as coke deposition and cracking during the process.^251^ Methane was converted to methanol supported on a MgO–ZrO_2_ matrix using nickel (Ni). By increasing metal dispersion and decreasing carbon deposition, strontium (Sr) improves catalytic efficiency and increases activity and stability during the bireforming of methane.^252^ Moreover, methane is converted to methanol by employing copper (Cu). An active-center-based catalyst (CuNC-600) for the partial oxidation of methane to methanol was found to have high metal loading of 17.7 wt% Cu. H_2_O_2_ was used as an oxidant, and this catalyst exhibited good mass activity and selectivity towards C_1_ liquid products.^253^

### 3.2 Bimetallic catalysts

The selective oxidation of methane to methanol was studied using palladium-based bimetallic catalysts, especially AuPd nanoalloys, supported on titanosilicate TS-1. Given that gold (Au) may boost the release of highly reactive oxygen-based radicals from metal surfaces, which accelerates the conversion process, these catalysts functioned effectively. The capacity of these catalysts to produce reactive oxygen species, which are necessary for the formation of methyl radicals and subsequent oxygenates, is correlated with their performance (Tables 6–9).^254^

**Table 6:** A comparison of various supported metal catalysts for the selective oxidation of methane to methanol using H_2_ and O_2_ (ref. 254)

Entry	Catalyst	CH_3_OH (µmoles)	CH_3_OOH (µmoles)	HCOOH (µmoles)	CO_2_ (µmoles)	Total products (µmoles)	Total oxygenates (µmoles)	CH_3_OH selectivity (%)	Oxygenate selectivity (%)	TOF (h^−1^)
1	Blank	0.06	0.00	0.00	0.45	0.51	0.06	11	11	—
2	AuPd/TiO_2_ (from ref. 8)	1.31	0.29	0.0	0.32	1.92	1.60	68	83	0.38
3	AuPd/TiO_2_	1.40	0.18	0.82	1.24	3.64	2.40	39	66	0.73
4	AuPd/ZSM-5	0.38	0.07	1.54	0.75	2.74	1.99	14	73	0.54
5	AuPdPt/TS-1	1.15	0.48	0.88	0.98	3.49	2.51	33	72	0.52
6	0.66 wt% AuPd/TS-1	0.48	0.00	0.11	0.52	1.11	0.59	43	53	1.68

**Table 7:** Effect of the Au : Pd ratio on the activity of AuPd/TS-1 catalysts^254^

Entry	Catalyst	CH_3_OH (µmoles)	CH_3_OOH (µmoles)	HCOOH (µmoles)	CO_2_ (µmoles)	Total products (µmoles)	Total oxygenates (µmoles)	CH_3_OH selectivity (%)	Oxygenate selectivity (%)	TOF (h^−1^)
1	0.66% Au	0.17	0.00	0.09	0.49	0.75	0.26	22	34	1.61
2	0.55% Au–0.11% Pd	0.22	0.00	0.04	0.46	0.72	0.26	30	36	1.37
3	0.44% Au–0.22% Pd	0.21	0.00	0.39	0.37	0.96	0.59	22	62	1.63
4	0.33% Au–0.33% Pd	0.48	0.00	0.11	0.52	1.11	0.59	43	53	1.68
5	0.11% Au–0.55% Pd	0.40	0.00	0.07	0.27	0.74	0.47	54	63	0.92
6	0.66% Pd	0.09	0.02	0.30	0.31	0.72	0.41	15	57	0.84

**Table 8:** Catalytic activity of the 0.33 wt% Au–0.33 wt% Pd/TS-1 catalyst for the *in situ* oxidation of methane with multiple reuse cycles^254^

Cycle	CH_3_OH (µmoles)	CH_3_OOH (µmoles)	HCOOH (µmoles)	CO_2_ (µmoles)	Total products (µmoles)	Total oxygenates (µmoles)	CH_3_OH selectivity (%)	Oxygenate selectivity (%)	TOF (h^−1^)
1	0.48	0.00	0.11	0.52	1.11	0.59	43.6	53	1.66
2	0.44	0.04	0.17	1.12	1.77	0.65	25.0	37	2.65
3	0.41	0.13	0.12	0.59	1.25	0.66	32.7	53	1.87

**Table 9:** Catalytic activity of 0.33% Pd–0.33% X/TS-1 (X = Au, Cu, Ni, Mn) catalysts towards the selective oxidation of methane *via in situ* H_2_O_2_ production

Catalyst	CH_3_OH (µmoles)	CH_3_OOH (µmoles)	HCOOH (µmoles)	CO_2_ (µmoles)	Total products (µmoles)	Total oxygenates (µmoles)	CH_3_OH selectivity (%)	Oxygenates selectivity (%)	TOF (h^−1^)
Pd	0.09	0.02	0.30	0.31	0.72	0.41	15	57	0.84
AuPd	0.48	0.00	0.11	0.52	1.11	0.59	43	53	1.68
CuPd	0.32	0.01	0.00	0.20	0.54	0.33	60	62	0.47
NiPd	0.14	0.00	0.46	0.63	1.23	0.60	11	49	1.05
MnPd	0.24	0.00	0.00	0.44	0.68	0.24	35	35	0.56

**Fig. 6:**
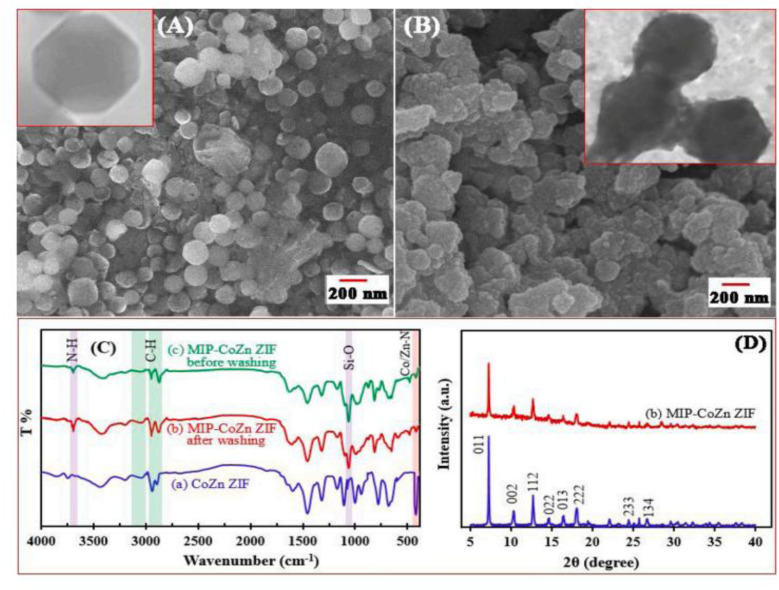
SEM images of CoZn ZIF (A) and MIP-CoZn ZIF (B); the insets show the corresponding TEM images. FT-IR (C) and XRD (D) spectra of CoZn ZIF and MIP-CoZn ZIF.^255^

Cobalt–zinc imidazolate frameworks (CoZn ZIFs) are bimetallic catalysts that can be utilized to convert methane to methanol. This framework increases the catalytic activity, which may result in more efficient reactions (Fig. 6).^255^

**Fig. 7:**
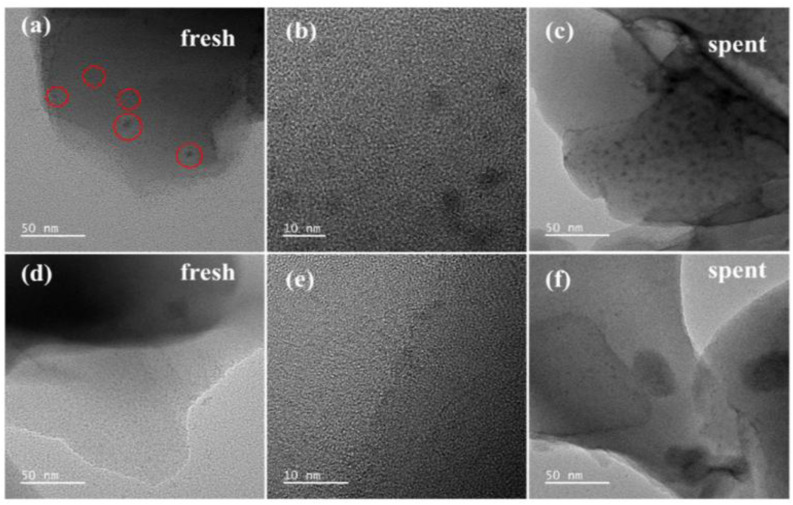
TEM images of fresh catalyst and the catalyst after three cycles: Cu_0.50_-MOR (a–c) and (d–f) Cu_0.50_Zn_0.35_-MOR.^257^

**Fig. 8:**
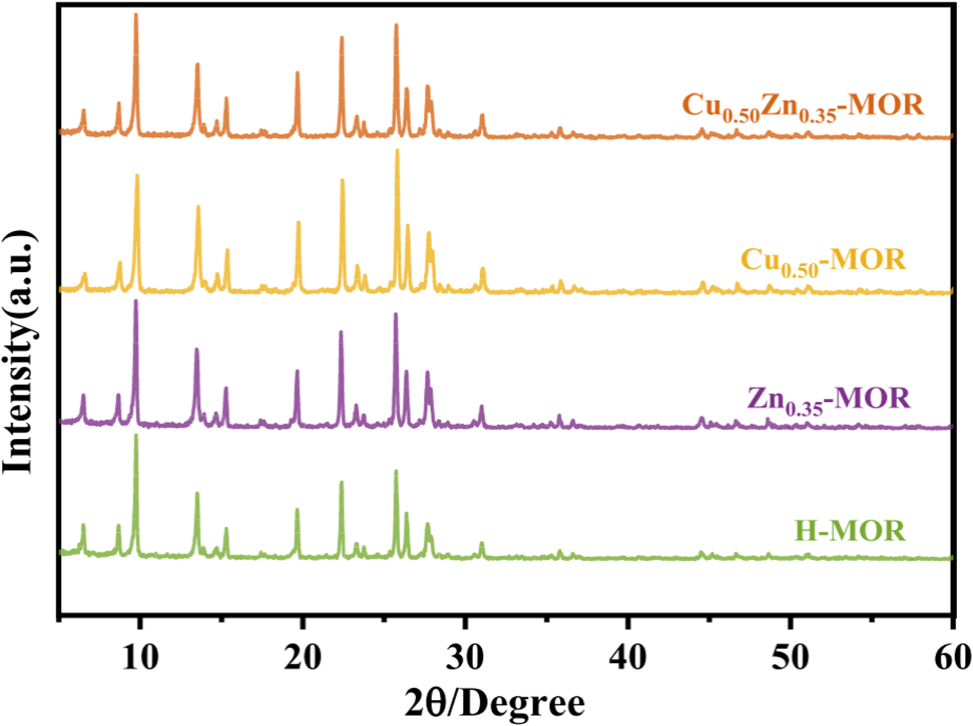
XRD patterns of various catalysts obtained using impregnation methods.^257^

AuPd and PdCu, in particular, are bimetallic dimers that aid in the transformation of methane into methanol. In comparison to monometallic systems, these dimers showed reduced activation barriers for methanol desorption and CH_4_ activation. They are potential candidates for direct methane-to-methanol conversion because they enhance the turnover frequency (TOF) and enable the activation of CH_4_ in both wet and dry settings.^256^ A bimetallic catalyst for the conversion of low-concentration methane to methanol was composed of a copper (Cu) and zinc (Zn) co-modified H-MOR zeolite. The catalyst exhibited increased stability and activity, yielding the maximum amount of methanol (71.35 µmol g_cat_^−1^ h^−1^). Zn was introduced into the zeolite framework to enhance the dispersion of Cu. This decreased the production of aluminum outside of the framework and encouraged the creation of active sites for the oxidation of methane (Fig. 7 and 8).^257^

NiCu/CeO_2_ is a bimetallic catalyst employed to convert methane to methanol. The addition of Cu to the Ni/CeO_2_ catalyst increased the C–H bond cleavage activation energy barrier but had little impact on O–H bond activation. This change favors methoxy formation over additional methyl species dehydrogenation, thereby improving the selectivity towards methanol generation.^258^ During biomass reforming processes, bimetallic catalysts, particularly Ni–Co, improve methane conversion rates, decrease coke production, and increase stability.^259^ Ni-, Cu-, and Pd-based systems are the main examples of bimetallic catalysts for the production of methanol. The synthesis of methanol is improved by these catalysts through higher activity and selectivity. Pd-based systems doped with In, Zn, or Ga exhibit notable improvements in performance, such as increased turnover frequency (TOF) and stability.^260^

#### 3.2.1 Noble metals

**Fig. 9:**
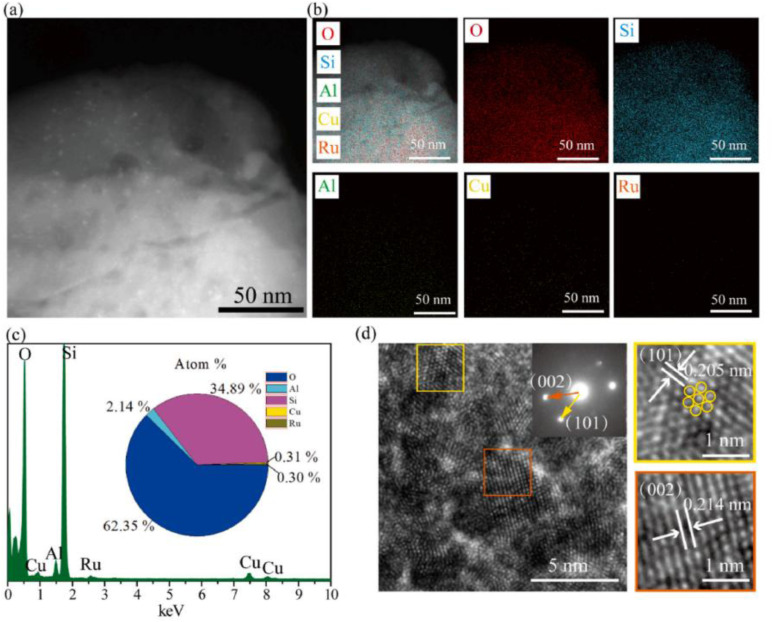
A TEM image (a), EDX elemental mapping images (b), the EDX surface scanning spectrum (c), and crystal lattice information (d) relating to a Ru/Cu-ex-MOR catalyst.^239^

**Fig. 10:**
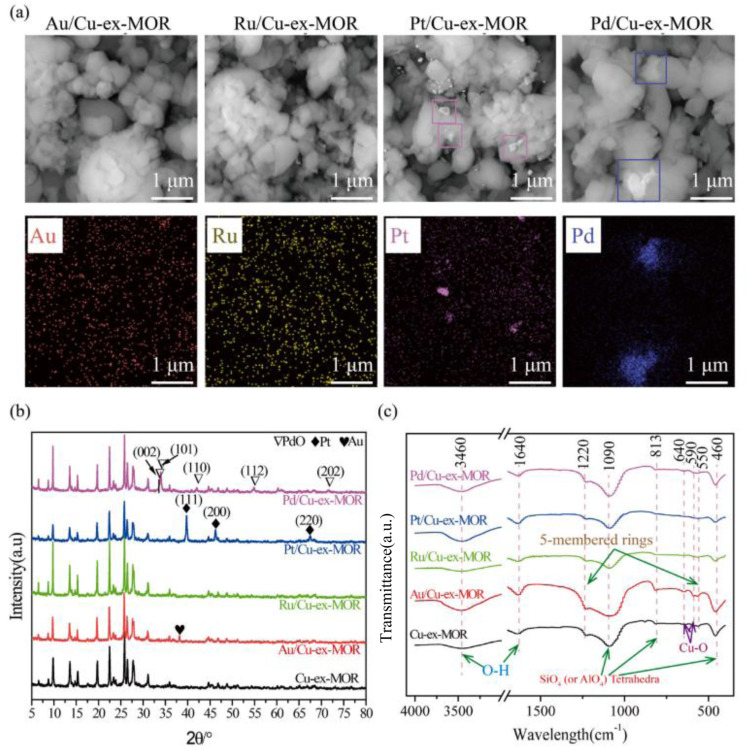
Structural characterization of noble-metal-stabilized Cu-MOR: (a) SEM imaging and EDS mapping of Au, Ru, Pt, and Pd/Cu-ex-MOR, (b) XRD patterns, and (c) FT-IR spectra.^239^

The process of converting methane into methanol using noble metals such as ruthenium (Ru) and gold (Au) has been studied (Fig. 9). The interaction of these metals improves the catalytic activity, which improves the efficiency of the oxidation of volatile organic compounds (VOCs). More specifically, compared to monometallic catalysts, a Au–Ru bimetallic system exhibits a substantial synergistic effect, which is essential for obtaining better catalytic efficiency.^261^ Noble metals such as Pd, Rh, and Ru supported on reducible oxide carriers (*e.g.*, FeO*x*, MnO*x*, NiO*x*, and SnO*x*) play a crucial role in C–H bond activation. In these systems, the noble metal typically serves as the primary site for C–H bond cleavage, while the reducible oxide support functions as an electronic promoter and facilitates hydrogen transfer through its ability to act as a hydrogen acceptor, oxygen donor, and oxygen-vacancy provider. Consequently, the most energetically favorable reaction pathway generally occurs at the metal–oxide interface, where synergistic interactions between the noble metal and the reducible support promote efficient C–H activation. This cooperative mechanism often results in enhanced catalytic activity and selectivity compared with either component acting independently.^258,262^ The noble metal palladium (Pd) was used in the process of turning carbon dioxide into methanol. Bimetallic catalysts based on Pd, especially those doped with metals such as Ga, have demonstrated increased catalytic activity and selectivity for methanol generation. When Ga was added to a Pd system, the electronic structure improved, and the maximum rate of methanol production was maximized, resulting in a considerable improvement in methanol selectivity to 66%.^260^ Methane was converted to methanol using ruthenium (Ru), gold (Au), platinum (Pt), and palladium (Pd). Ru considerably increased the methanol production to 157.36 µmol g_cat_^−1^ h^−1^ compared to 12.89 µmol g_cat_^−1^ h^−1^ for the unaltered catalyst. Additionally, Au and Pt increased the yields, whereas Pd agglomeration decreased the yield of methanol and hindered mass transfer and access to active sites (Fig. 10).^239^

Gold (Au) and palladium (Pd) have been used to convert methane into methanol. By facilitating the release of reactive oxygen-based radicals, which are essential for the oxidation process, Au improves the catalytic activity of Pd.^254^ Ruthenium (Ru), palladium (Pd), and platinum (Pt) have been used to convert methane to methanol. These metals improved CO removal and methanol oxidation efficiency by increasing the catalytic activity and stability.^263^ In the process of converting methane to methanol, metals such as Rh, Ru, Pd, Pt, and Ir enhance the catalytic activity and resistance to carbon production.^264^ In addition, the anti-sintering properties of catalysts with noble metals such as gold (Au), ruthenium (Ru), platinum (Pt), and palladium (Pd) increase the electronic structure and maximize the efficiency of the methane-to-methanol conversion process during dry reforming of methane.^265^ The process of using palladium (Pd) and gold (Au) for methanol production was studied, and adding Au improves the productivity and selectivity of methanol obtained at 2.5% Au loading in Pd–Au nanoparticles supported on carbon nanotubes.^266^

#### 3.2.2 Transition metals

**Fig. 11:**
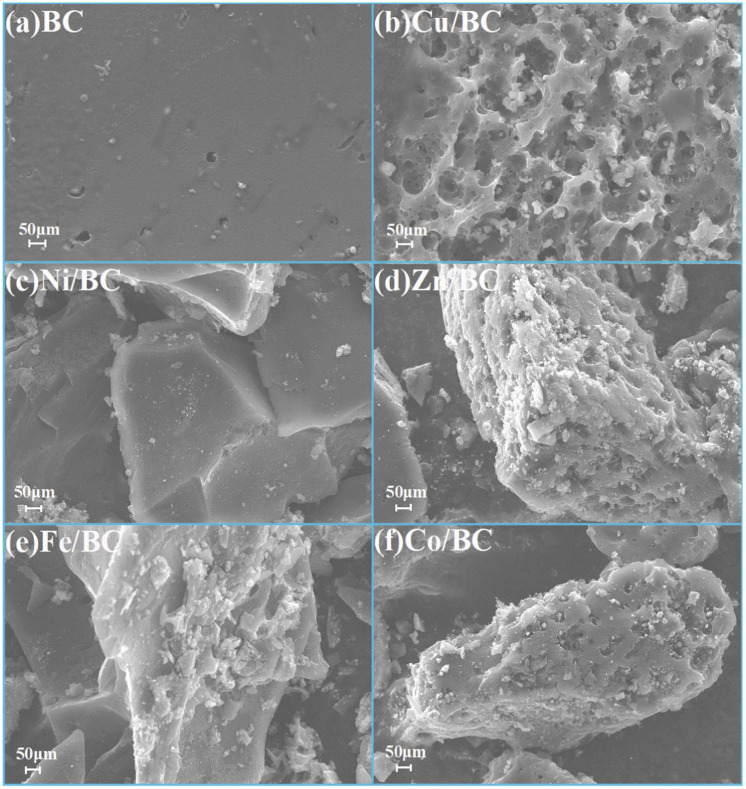
SEM images of BC and calcined catalysts. (a)BC, (b) Cu/BC, (C) Ni/BC, (d) Zn/BC, (e) Fe/BC and (f) Co/BC.

**Fig. 12:**
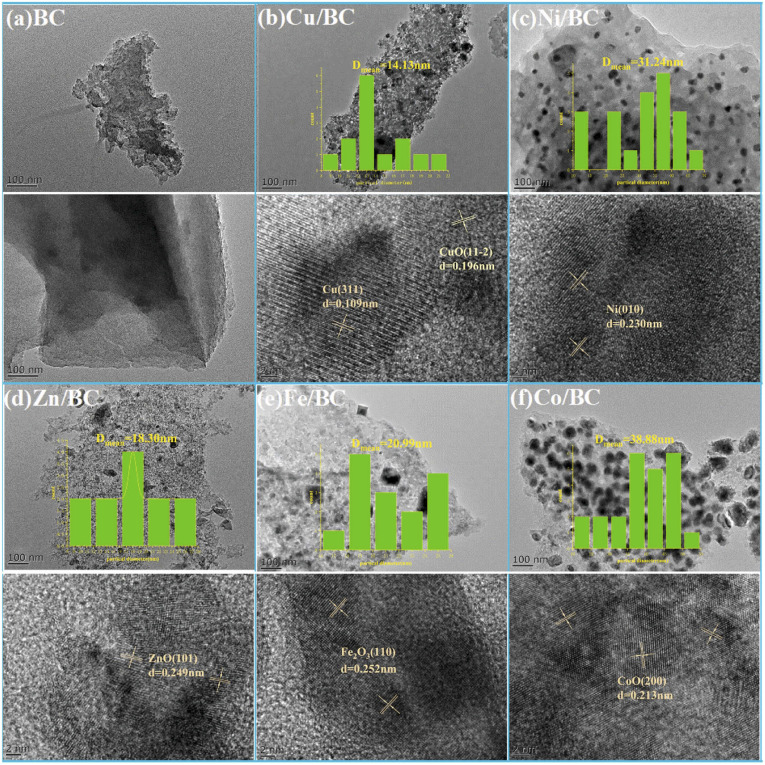
HRTEM images of BC and calcined catalysts. (a) BC, (b) Cu/BC, (C) Ni/BC, (d) Zn/BC, (e) Fe/BC and (f) Co/BC.

Methane has been converted to methanol using several transition metals, including copper (Cu), nickel (Ni), zinc (Zn), iron (Fe), and cobalt (Co). Cu had the greatest activity and stability among them, yielding 686.92 µmol of methanol/g Cu/h under ideal conditions. By enabling the activation of methane while promoting the synthesis of methanol by removing oxygen-containing functional groups from charcoal (BC), the addition of transition metals improved catalytic activity (Fig. 11 and 12).^205^

**Fig. 13:**
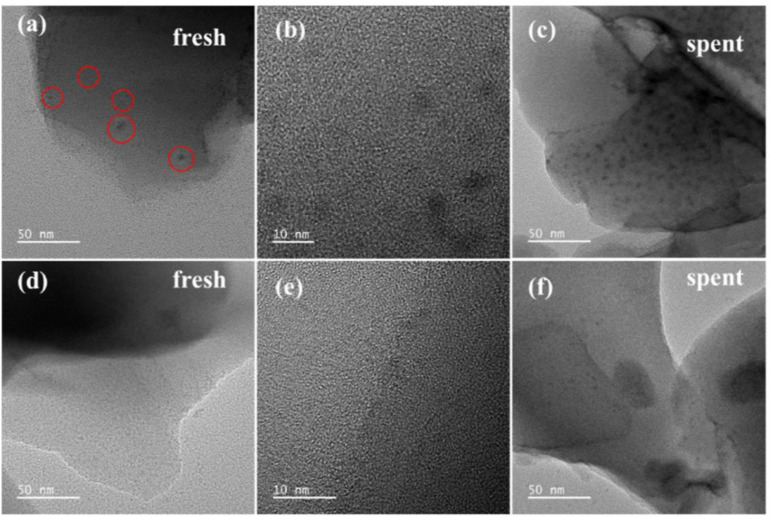
TEM images of fresh catalyst and the catalyst after three cycles: Cu_0.50_-MOR (a–c) and (d–f) Cu_0.50_Zn_0.35_-MOR.^257^

Copper (Cu) and iron (Fe) metals were utilized to convert methane to methanol. Compared to monometallic catalysts, the bimetallic catalyst CuFe supported on g-C_3_N_4_ significantly increases the methanol selectivity because Cu–O–Fe active sites are formed, facilitating the effective dissociation of H_2_O_2_ into hydroxyl radicals and increasing methanol yields.^267^ The transition metal nickel (Ni), more precisely in the form of NiFe alloy nanoparticles, converts methane to methanol. The addition of iron (Fe) considerably improves the catalytic efficiency of Ni-based catalysts.^268^ Copper (Cu), palladium (Pd), gold (Au), iron (Fe), nickel (Ni), cobalt (Co), silver (Ag), and zinc (Zn) are among the metals that can be used to convert methane to methanol. The results have shown that in both dry and wet environments, Cu, Pd, and Au dimers have promising turnover frequencies (TOFs), with Cu demonstrating notable activity owing to its capacity to alter oxidation states and promote reaction processes.^256^ When converting methane to methanol using copper (Cu) and zinc (Zn), Zn promotes the dispersion of Cu and stabilizes the catalyst, resulting in better methanol yields when Cu is added to the catalyst. Cu increases the catalytic activity. The bimetallic catalyst Cu_0.50_Zn_0.35_-MOR, in particular, outperformed monometallic catalysts, achieving a maximum methanol production of 71.35 µmol gcat ^−1^ h^−1^ (Fig. 13).^257^

**Fig. 14:**
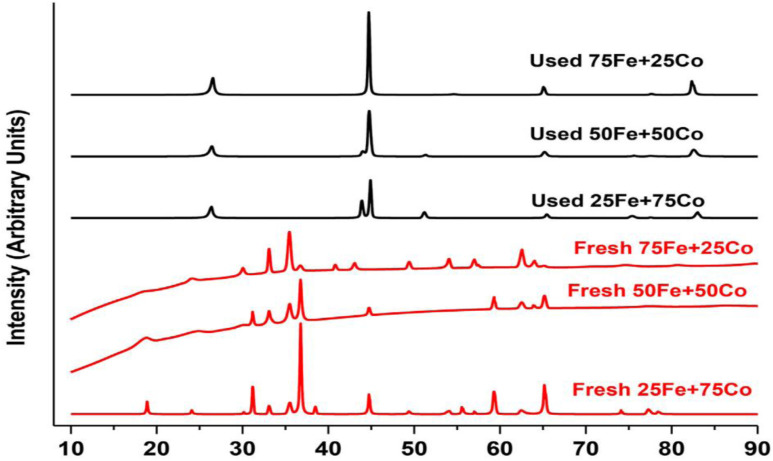
XRD patterns of fresh and used mixed oxide catalysts.^244^

**Fig. 15:**
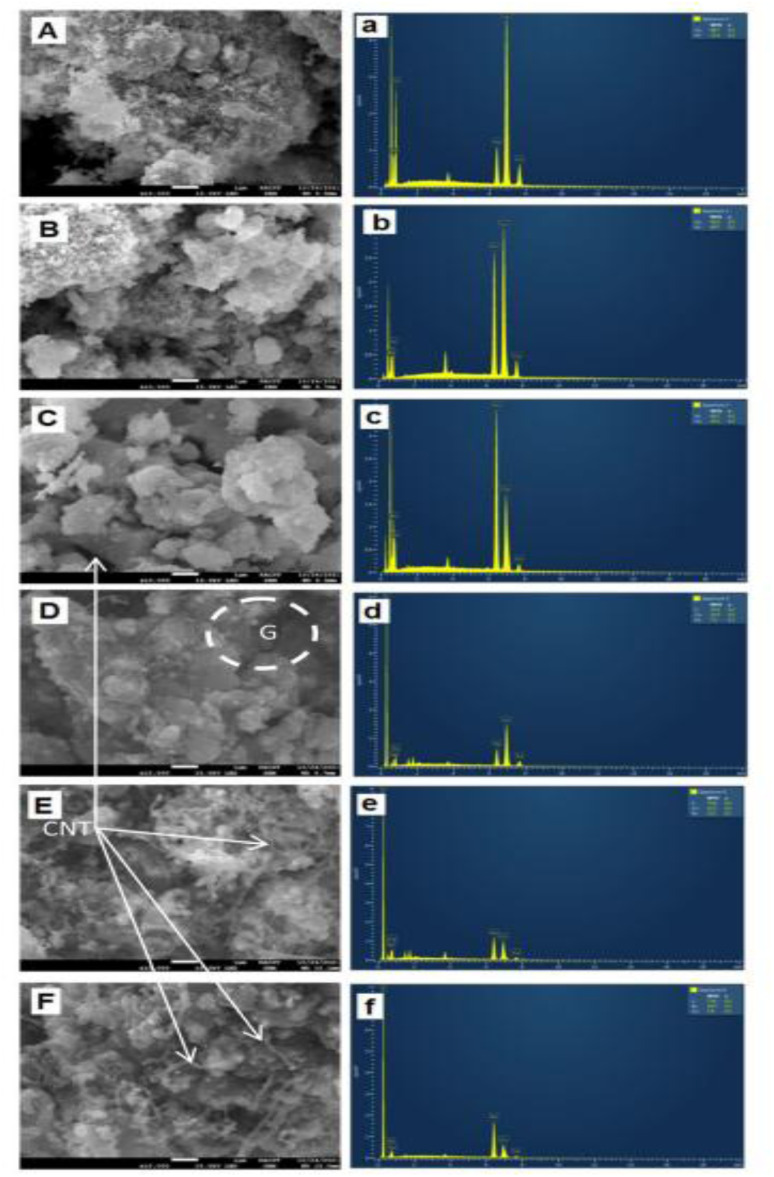
SEM and EDX analysis of fresh (A–C) and used (D–F) 25Fe + 75Co, 50Fe + 50Co, and 75Fe + 25Co catalysts.^244^

**Fig. 16:**
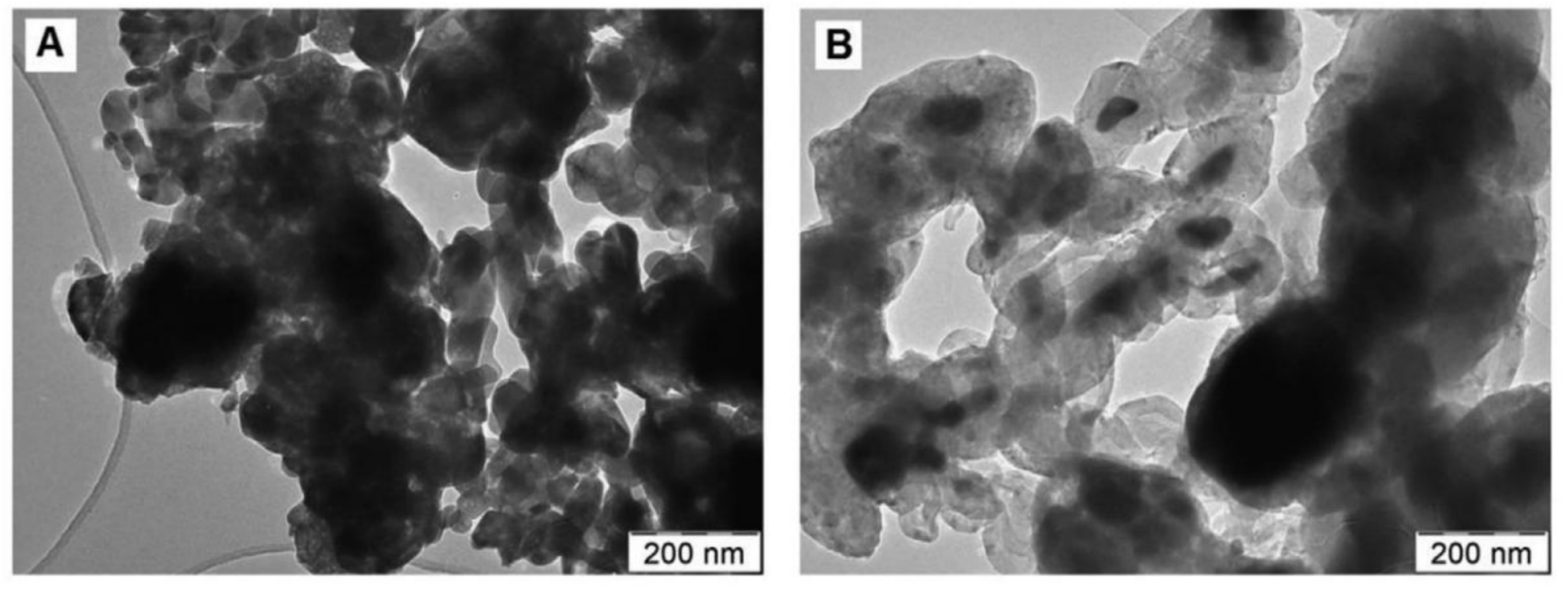
TEM images of fresh (A) and used (B) 50Fe + 50Co catalysts.^244^

The transition metal Mo has been studied in the methanol production process from methane. A high yield of 754 µmol g_Cu_^−1^ has been demonstrated for the efficient production of methanol from copper, specifically, in the form of Cu-ZSM-5. However, only 52% of the adsorbed methane was converted to methanol. With MoO_3_/Fe_2_O_3_ catalysts, molybdenum and iron (Fe) jointly produced 80% selectivity for methanol and formaldehyde with 3.5% methane conversion.^83^ Nickel (Ni) has been used, particularly in the form of Ni/Al_2_O_3_ and Ni–Cr/Al_2_O_3_ catalysts, to convert methane to methanol. Higher hydrogen yields and carbon synthesis were achieved using chromium (Cr) as a promoter, which also greatly enhanced the metal dispersion and reduction behavior. This also increased the catalytic efficiency and stability of Ni-based catalysts during methane decomposition.^269^ Iron (Fe), nickel (Ni), and cobalt (Co) were employed to convert methane to methanol. The non-filled 3d orbitals of these metals aid in the dissociation of methane molecules, consequently strengthening the catalytic activity and making them effective. The results demonstrated that Co–Fe mixed oxide catalysts, especially those with a 50Fe : 50Co ratio, performed better catalytically and provided noticeable carbon and hydrogen yields during the decomposition of methane (Fig. 14–16).^244^

Ni and its bimetallic combinations (Ni–M, where M = Co, Cu, and Fe) are transition metals used in a conversion process that produces methanol. The catalytic activities of these catalysts differed, with Ni–Fe demonstrating efficient conversion capabilities with a turnover frequency (TOF) of approximately 35 × 10^−3^ s ^−1^. These metals are necessary for the synthesis of methanol because they improve the reaction efficiency and selectivity towards the creation of methane.^270^ Palladium (Pd) and copper (Cu) complexes are used to convert methane to methanol. Pd lowers the energy barriers in the hydrogenation phase and increases hydrogen dissociation, which facilitates the development of methanol.^271^ Catalysts made of iron (Fe) and copper (Cu) are used to convert methane into methanol. These metals improve the selectivity and catalytic activity for methanol synthesis, especially when coupled. High productivity and selectivity were demonstrated by a Cu–Fe/Al_2_O_3_ catalyst, particularly in the presence of N_2_O and H_2_O oxidants.^272^

## 4 Sources of oxidant for methane-to-methanol conversion

Several oxidants have been investigated for methane-to-methanol conversion, with molecular oxygen (O_2_), water (H_2_O), hydrogen peroxide (H_2_O_2_), and nitrous oxide (N_2_O) being the most commonly reported. Their effectiveness depends on the catalyst system, reaction conditions, and desired methanol selectivity.^273^ Molecular oxygen (O_2_) is the most widely used oxidant because of its availability and strong oxidation capability. O_2_ has been successfully employed with Cu/Ti-ZSM-5 catalysts under photothermal conditions, improving the catalytic performance of methane oxidation to methanol (W. Wang, Hao, *et al.*, 2024). Similarly, O_2_ plays an essential role in the low-temperature oxidation process over Cu-MOR/g-C_3_N_4_ photocatalysts, resulting in enhanced methanol yields.^274^ O_2_ has also been investigated in copper-based catalysts and photocatalytic systems, where it promotes the formation of reactive oxygen species and improves methane activation and conversion efficiency.^239^ Furthermore, molecular oxygen has demonstrated effectiveness in selective oxidation reactions and continuous gas-phase selective oxidation of methane to methanol.^199^ Water (H_2_O) is considered a mild, clean, and economical oxidant for methane-to-methanol conversion. Water has been reported as a primary oxidant in photocatalytic systems, where the generation of hydroxyl radicals (˙OH) facilitates methane oxidation under light irradiation.^275^ Water can improve methanol selectivity by suppressing overoxidation and has been utilized in continuous methane-to-methanol conversion processes.^276^ In some systems, water acts together with small amounts of O_2_, where O_2_ provides stronger oxidation ability while water contributes to selective methanol formation.^15,277^ The versatility of water as an oxidant has also been demonstrated in GaN-based photocatalytic systems, which selectively produce methanol in the absence of O_2_ and formic acid when O_2_ is present.^278^ Moreover, O_2_ and H_2_O are both affordable and widely available oxidants, and H_2_O_2_ can be generated in situ from O_2_ under suitable conditions.^188^ Hydrogen peroxide (H_2_O_2_) is frequently employed as an oxidant because it readily generates hydroxyl radicals that enhance methane activation and methanol production. H_2_O_2_ has been extensively used in photocatalytic methane conversion under mild or ambient conditions, where its decomposition produces reactive ˙OH species that improve oxidation efficiency.^279^ H_2_O_2_ is often used together with O_2_ in photocatalytic systems and has been shown to enhance methane conversion efficiency and selectivity toward methanol.^211^ In addition, H_2_O_2_ has been investigated as an effective oxidant in various methane oxidation processes because of its ability to promote partial oxidation while limiting deep oxidation products.^172^ Nitrous oxide (N_2_O) is another important oxidant for direct methane-to-methanol conversion. N_2_O can generate highly active oxygen species at relatively low temperatures, thereby improving methane activation and methanol formation efficiency.^280^ N_2_O has been successfully employed in gas-phase systems using zeolite-supported catalysts, particularly copper ion-exchanged mordenite (Cu-ex-MOR) catalysts.^187^ It has also been investigated with Fe-ZSM-5 catalysts, where it promotes partial methane oxidation while minimizing overoxidation.^198^ High methanol yields have additionally been reported with Cu/BEA zeolite catalysts using N_2_O as the oxidant.^281^ Besides these major oxidants, other oxygen-containing species, including OOH*, O*, OH*, NOx, and air, have also been explored for methane-to-methanol conversion. These oxidants contribute to the formation of reactive oxygen intermediates that facilitate methane activation and selective oxidation to methanol.^195,205,282^ Overall, O_2_, H_2_O, H_2_O_2_, and N_2_O remain the dominant oxidants investigated for methane-to-methanol conversion, each offering distinct advantages in terms of activity, selectivity, cost, and reaction conditions.^283^

### 4.1 Oxygen as an oxidant for methane-to-methanol conversion

The conventional method of converting methane into methanol involves the reaction of methane with oxygen (O_2_) as an oxidant at temperatures of approximately 250 °C to produce methanol and Cu^+^. Cu^+^ is reoxidized during methanol desorption with water vapor in this procedure.^191^ Oxygen serves as an oxidant to convert methane to methanol, while water-derived highly oxidizing ˙OH radicals aid in activating methane, resulting in ˙CH_3_ radicals that subsequently transform into methanol.^284^ To convert methane to methanol and increase the production of methanol during cyclic operations, oxygen (O_2_) has been used as an oxidant. In subsequent reactions at 400 °C, the methanol output gradually increased while preserving the Cu^2+^ oxidation state, which is essential for catalytic activity.^285^ Methane was directly converted to methanol using oxygen as an oxidant. The impact of the CH_4_/O_2_ ratio was investigated, and it was found that lower ratios enhanced methane conversion while limiting methanol selectivity owing to possible overoxidation.^188^ The thermochemical dissociation of carbonate ions (CO_3_^2−^) electrochemically adsorbed on the Fe_2_O_3_ catalyst provides an oxygen supply for the conversion of methane to methanol, producing active oxygen (O*) that accelerates the reaction.^18^ O_2_ has been demonstrated to be a promising oxidant for the conversion of dinuclear copper (Cu_2_) species into dicopper-oxo (Cu_2_O_2_) species, indicating thermodynamic and kinetic favorability for the oxidation of methane to methanol inside the MIL-53(Al) framework.^286^ As the active oxygen species for methane conversion, surface lattice oxygen (O_s-latt_) is crucial for coupling surface hydroxyls with methane radicals and oxidizing methane to methanol through several different pathways.^287^ Syngas is developed when methane undergoes partial oxidation, with oxygen acting as the oxidant. This approach enables a low oxygen-to-methane molar ratio and effective heat recovery, both of which are advantageous for subsequent methanol synthesis procedures.^288^ In photocatalytic systems, O_2_ molecules directly contribute to the partial oxidation of CH_4_; oxygen plays a critical role as an oxidant in the conversion of methane to methanol, improving the conversion efficiency.^289^ The selective photocatalytic conversion of methane to methanol requires oxygen as an oxidant. The activation of methane is facilitated by the presence of oxygen, which additionally encourages the production of reactive oxygen species that are necessary for efficient C–H bond activation during photoexcitation.^290^ Low-concentration methane was effectively partially oxidized to methanol using airborne oxygen as the oxidant, exhibiting a cost-effective conversion method.^279^ Under mild conditions, oxygen (O_2_) is used as a gentler oxidant to produce reactive oxygen species (ROS), particularly hydroxyl radicals (˙OH), which help activate and convert methane to methanol.^21^ Methane was converted to methanol in stages using oxygen as the oxidant. The method produces active copper species that help convert methane at lower temperatures by activating oxygen over copper-exchanged mordenite at high temperatures, typically at approximately 450 °C.^197^ Under ambient conditions, methane (CH_4_) was converted to methanol (CH_3_OH) using molecular oxygen (O_2_) as an oxidant. Achieving high selectivity of 90.65% for methanol production, the presence of O_2_ increased the formation of reactive radicals.^278^

## 5 Different reactors used for methane-to-methanol conversion

**Fig. 17:**
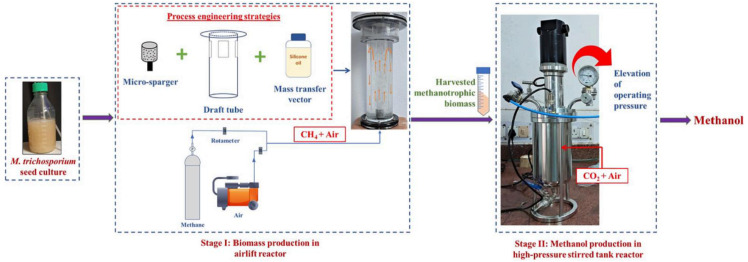
Schematic diagrams of an airlift reactor and a high-pressure stirred tank reactor.^292^

Methane was converted to methanol using a high-pressure stirred tank reactor and an airlift reactor (Fig. 17). The first stage involves sequestering methane in an airlift reactor fitted with a draft tube and microsparger to produce methanotrophic biomass. The addition of a mass transfer vector, such as silicone oil, improves the solubility and mass transfer in the reactor. In the second step, biomass is used as a biocatalyst in a high-pressure stirred tank reactor to transform methane into methanol.^291^

**Fig. 18:**
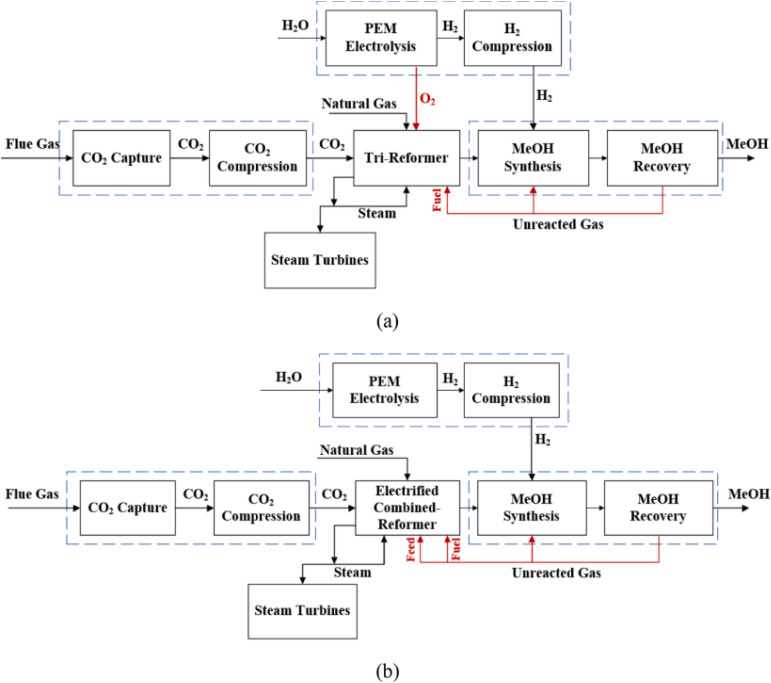
(a and b) Simplified diagrams showing the TRM and E-CRM processes.^295^

The process of converting methane into methanol has been carried out in a continuous flow reactor. A Cu_1_/γ-Al_2_O_3_ single-atom catalyst (SAC) was evaluated in a continuous process in which the reactor was continuously supplied with a reaction gas combination that included steam and methane. The reactor settings included pretreatment in helium flow at 400 °C, cooling to 200 °C for the reaction, and online mass spectrometry detection of the products.^293^ The use of a small fixed-bed reactor was established to convert methane to methanol. This reactor consists of a 600 mm high, 10 mm diameter reaction tube with a 300 mm-long liner tube. The catalyst was secured in the reaction setup using a layer of quartz cotton, and the reaction was carried out at 320 °C. By employing a combination of oxygen and water vapor as co-oxidants, the reactor helps methane undergo partial oxidation to methanol.^239^ In a fixed-bed stainless-steel reactor, the catalyst was loaded onto a quartz wool support after being diluted with H-MOR for the continuous oxidation of methane into methanol. A mass flow controller regulated the gas flow rates, whereas a syringe pump controlled the flow of liquid water. Water condensation is avoided by keeping the gas lines heated to temperatures above 110 °C.^294^ Continuous gas-phase selective oxidation of methane to methanol (SOMM) was carried out using a fixed-bed reactor. To investigate the catalytic activity, 50 mg of catalyst powder was placed in a quartz tube and activated using artificial air. Methane, water vapor, and synthetic air were pumped into the reactor, which was heated to 280 °C, to accomplish the conversion process.^205^ A modified electric combination reforming reactor (E-CRM) was used to convert methane into methanol. This reactor utilizes electrification to improve the effectiveness of the reforming process. Four TRM reactor configurations were also represented by a one-dimensional heterogeneous model, which included a conventional multi-tubular fixed-bed reactor and three membrane reactors that used O_2_, CO_2_, or H_2_O side-feeding techniques (Fig. 18).^295^

**Fig. 19:**
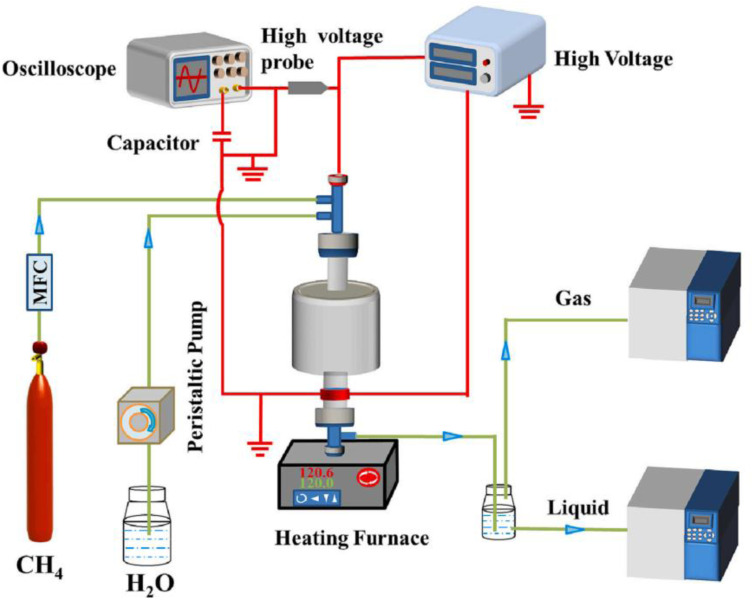
A diagram of the DBD plasma methane conversion experimental process.^275^

The process of converting methane into methanol using a dielectric barrier discharge (DBD) plasma reactor is described. This reactor has a medium- and high-voltage electrode set up with a discharge length of 20 mm and a discharge gap of 3 mm. The medium was a quartz glass tube, and the reactor was loaded with Cu-mordenite catalyst grains. An absorption bottle filled with deionized water collects the liquid product after monitoring the methane flow using a mass flow controller (Fig. 19).^275^

**Fig. 20:**
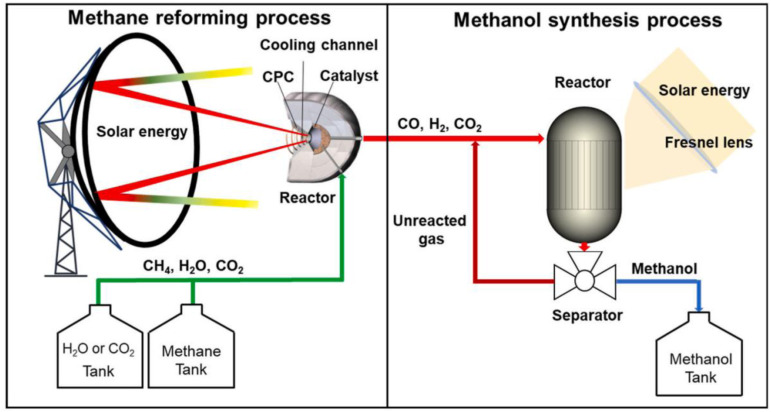
A schematic diagram of a solar methane-to-methanol experimental platform.^297^

The process of transforming methane into methanol in a tubular stainless-steel fixed-bed reactor was studied. A thermocouple was used to measure the temperature of the material inside the reactor, which had an inner diameter of 9 mm. A water bath was used to inject water vapor into the reactor, and a system was incorporated to control the flow rates of gases, including nitrogen, oxygen, and methane. To track the product concentrations, the reactor was linked to an online FTIR gas analyzer.^197^ The photocatalytic conversion of methane to methanol was performed using two different types of reactors. Combining a suspension of deionized water and GaN powder, a small-scale catalytic performance test was carried out in an airtight quartz reactor using a 12 mL quartz tube. Large-scale experiments were conducted using a 120 mL chamber reactor with a quartz window and a suspension of GaN powder and deionized water. To accelerate the reaction, both reactors were evacuated and operated under controlled circumstances.^278^ Different reactor setups for methanol production from methane have been used. These include fixed-bed reactors, which are frequently used in large-scale processes; fluidized-bed reactors, which are characterized by high heating rates and short residence times; batch reactors, which are straightforward but have challenges with heat transfer; continuous stirred tank reactors (CSTR), which provide continuous operation and improved mixing; and micro-plasma reactors, which facilitate single-step conversion with high selectivity.^195^ Methane was converted to methanol in a dielectric barrier discharge (DBD) reactor. This reactor operates on plasma filled with a mixture of oxygen and methane, produced by a 32 kHz AC high-voltage power source with a voltage range of 12–20 kV. The copper electrode is a component of the DBD reactor setup that has a major impact on the yield and selectivity of methanol.^296^ For the photocatalytic methane oxidation reaction, a 100 mL top-irradiated high-pressure reactor (YZPCR, Shanghai Yanzheng Experimental Instrument Co., Ltd) was used. To enable the reaction under regulated conditions, the reactor was operated in combination with a xenon lamp source. To establish the ideal conditions for the conversion process to methanol, the reactor was additionally injected with oxygen and methane and then purged with ultra-pure oxygen.^21^ Methane is converted to methanol using a fixed bed reactor, in which deionized water is sent to a preheating pipeline to produce water vapor, which is subsequently combined with streams of methane gas and air in a mixing chamber before entering the reaction tube, where partial oxidation occurs.^279^ A two-step solar-powered methane-to-methanol conversion method has also been used. The solar methane reforming module, the first reactor, uses a dish concentrator to convert methane into syngas while operating at high temperatures (750–1000 °C). The second reactor is a solar methanol synthesis module that uses Fresnel lens concentrators to convert syngas into methanol at lower temperatures (220–280 °C). This integrated system effectively drives the synthesis and reformation processes using concentrated solar electricity (Fig. 20).^297^

**Fig. 21:**
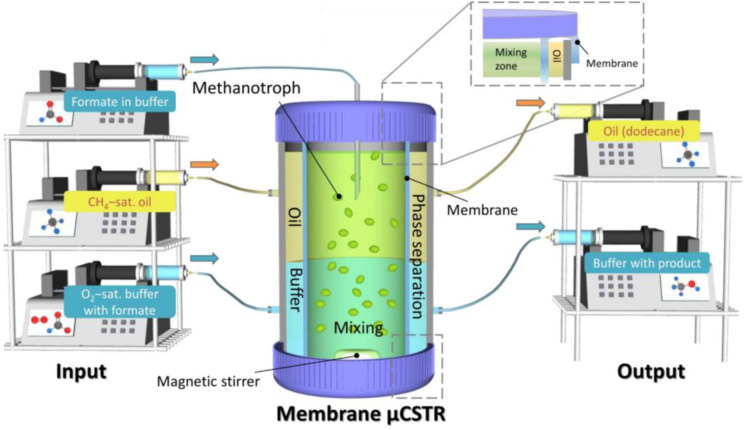
A schematic diagram of a spontaneous phase-separable membrane µCSTR system.^299^

An isopotential microchannel reactor with three isothermal phases improves the conversion efficiency of microchannel reactors for the conversion of methane to methanol. Furthermore, the effectiveness of Pt/Al_2_O_3_-coated microchannel reactors for methane steam reforming has been explored.^298^ Conversion of methane to methanol using a membrane microcontinuous stirred-tank reactor (µCSTR) has been investigated (Fig. 21). This reactor overcomes the drawbacks of batch reactors through improved mass transfer, continuous operation, and effective product separation by spontaneous phase separation. The µCSTR is an excellent option for this bioconversion process because it prevents the loss of whole-cell biocatalysts and promotes the recycling of unreacted reactants.^299^

Using dielectric barrier discharge (DBD) plasma, a type of non-thermal plasma reactor, to convert methane to methanol has been investigated. This study is part of the wider exploration of plasma catalysis in methane conversion technologies. The DBD plasma reactor is renowned for its capacity to improve methanol selectivity through the use of particular catalysts, including metal oxides, which are designed to promote the synthesis of organic oxygenates while minimizing undesirable byproducts.^8^ The integrated mixer-reactor-heat exchanger microtube scalable system is excellent for the non-catalytic direct partial oxidation of methane to methanol. To produce methanol selectively with per-pass yields higher than 8% and selectivities above 70%, this system enables the control of five essential parameters: temperature, pressure, residence duration, wall inertness, and the methane : air ratio. The process efficiency is increased by protecting the inner tubes using coating techniques in conjunction with microreactor technology.^300^ A fixed-bed reactor, also known as R1, was used to convert methanol to methane. After undergoing preheating, methanol enters this reactor in gaseous form, acting as a solar receiver. Synthesis gas, which is then processed to yield methane, originates from methanol. To ensure full conversion in the reaction cycle, the system incorporates a flash separator to recycle unreacted methanol.^301^ A counterflow moving bed reactor can convert methane into syngas, which is the basis for methanol synthesis. This reactor uses non-premixed fluxes of oxidant gas, steam, and methane while operating in filtration combustion mode. To improve energy efficiency and conversion rates, a solid granular heat carrier is used to separately preheat the reactants.^288^

### 5.1 Hydrothermal reactor systems

A three-dimensional spherical particle random-packed bed is a defining feature of the tubular hydrothermal packed-bed reactor model. The reactor has a packed bed height of 10 mm and measures 24 mm in length and 5 mm in radius. By enhancing the catalyst porosity and addressing internal diffusion restrictions, this structure improves the catalytic performance by optimizing the fluid flow and mass transfer.^39^ A microcoil tubular reactor was used in a continuous flow hydrothermal synthesis (CFHTS) technique to produce MoVNbTeO_*x*_ catalysts. By facilitating rapid synthesis at 300 °C and 10 MPa, this system significantly reduces the preparation time from 48 h to 10 min compared to traditional approaches, improving the catalytic performance and scalability of the process.^302^

**Fig. 22:**
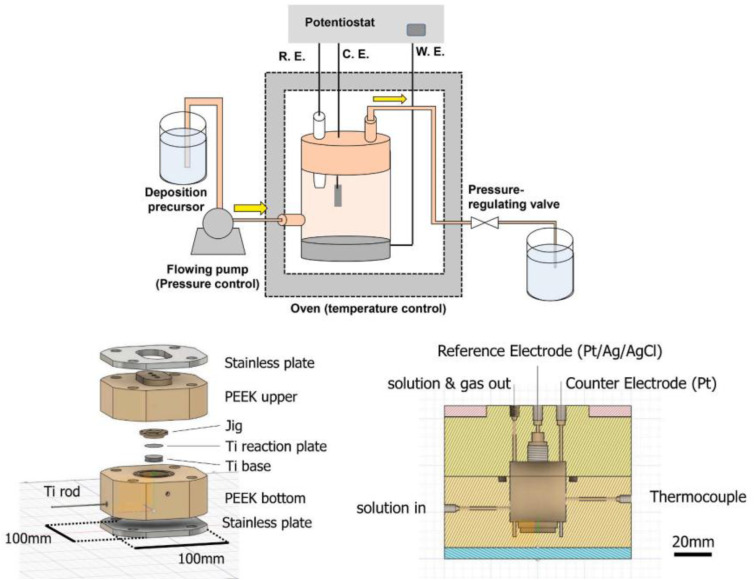
A schematic diagram of the hydrothermal electrochemical flow reactor showing (top) the system's overall structure and (bottom) the reactor's specific composition.^304^

A medium-sized palm oil mill incorporates pilot-scale hydrothermal treatment (HTT) equipment that can process 15 tons of wet EFB per hour. The energy and liquid sources used by the system were waste heat and condensate water. The treatment variables were a fixed liquid-to-solid ratio of 1.5 : 1, temperatures of 50, 60, 70, and 80 °C, and periods of 0, 20, and 30 min. By reducing inorganic pollutants, especially potassium, this configuration attempted to improve the characteristics of EFB fuel and minimize fouling during combustion.^303^ By independently regulating the temperature, pressure, and potential, a hydrothermal electrochemical flow reactor (HEFR) overcomes the disadvantages of conventional systems in which the temperature and pressure are correlated (Fig. 22). By adjusting the temperature and pressure, this reactor improves the electrochemical water oxidation activity and enables the exact electrodeposition of manganese oxides, improving the crystallinity and morphology.^304^

**Fig. 23:**
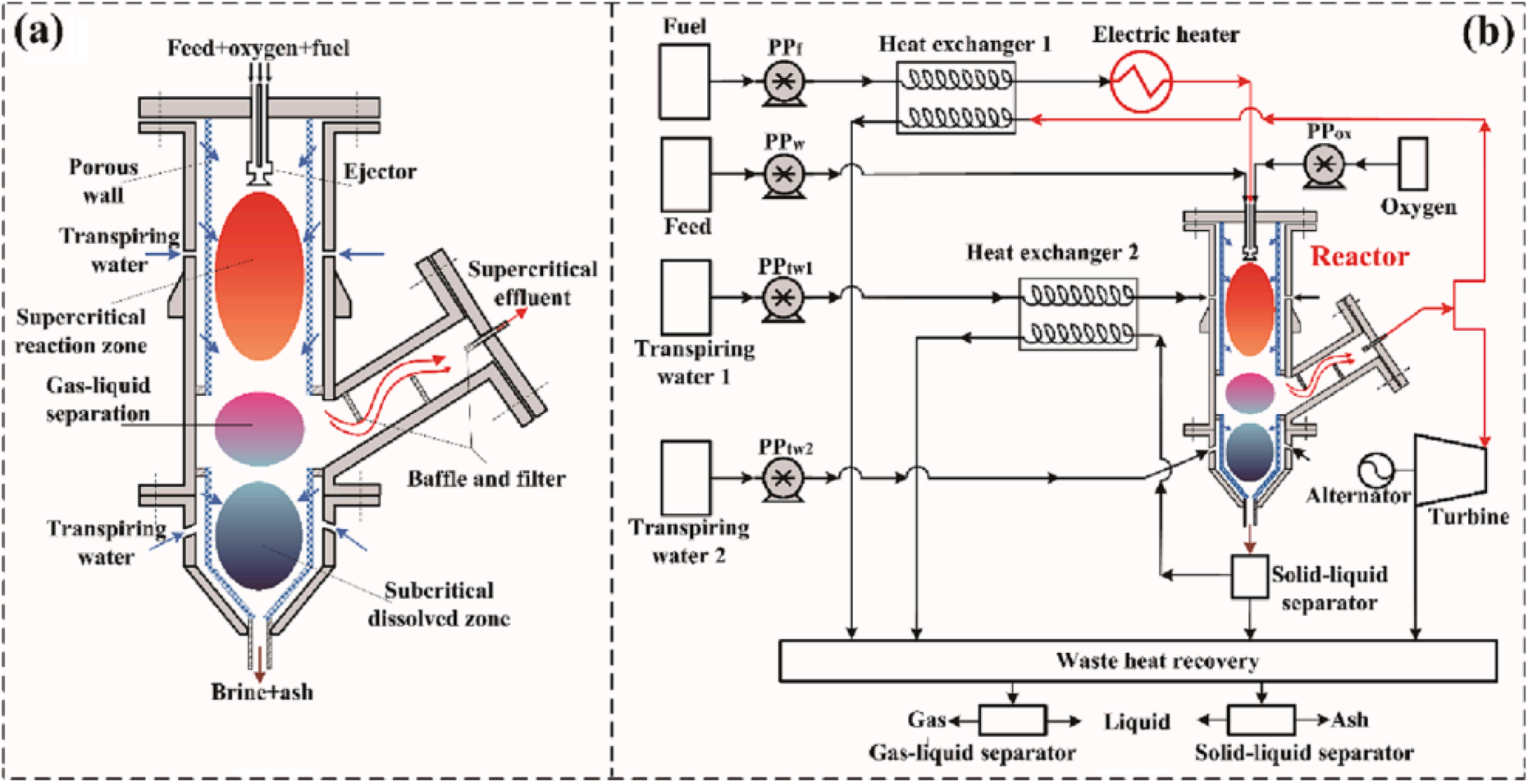
(a) The structure of a hypothetical Y-shaped SCWO reactor with a hydrothermal flame and (b) the energy recovery and utilization system needed to handle feedstocks with a high solid content.^305^

A new Y-shaped reactor for the supercritical water oxidation (SCWO) of wet organic waste that combines the ideas of hydrothermal flames and transpiring wall reactors (TWR) was developed (Fig. 23). This design makes it feasible to extract uncooled supercritical fluids for power generation and to effectively separate gases and solids. Complete organic degradation under hydrothermal flames becomes conceivable by the bottom cooling zone and upper reaction zone of the reactor system, which are shielded by high-temperature transpiring water.^305^

**Fig. 24:**
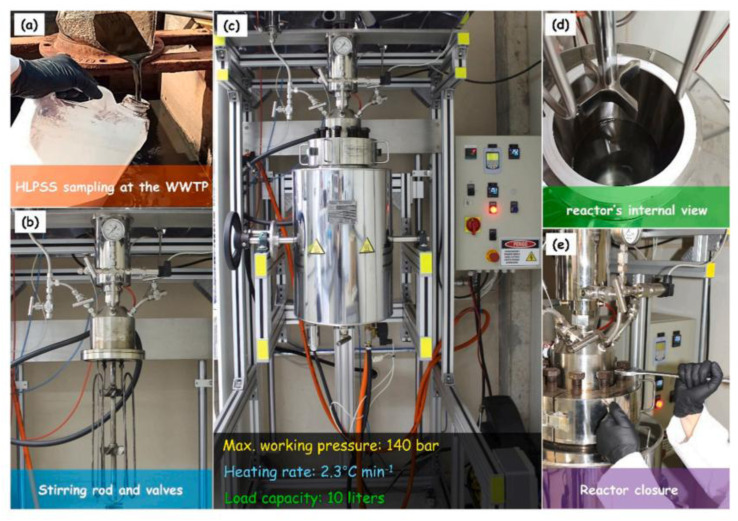
(a) HLPSS sampling in a WWTP; and (b–e) specifications for scalable HTC experiments in a high-pressure reactor.^307^

The hydrothermal reactor had a T-junction configuration and operated continuously at high pressure, with an output, a branch inlet for materials and cold water, and a main inlet for preheated water. The architecture of the reactor facilitates the analysis of residence time distribution (RTD) characteristics, which are essential for understanding material dispersion and fluid mixing under different flow conditions.^306^ An electrical resistance heating device and an axial mechanical stirrer are characteristics of a 10-liter stainless-steel high-pressure reactor (type 304). With only 30% of its capacity being used, the reactor was constructed with operator safety in mind, and it could sustain autogenic pressure between 6 and 16 bar. To ensure effective hydrothermal carbonization of sewage sludge, the system featured a cooling jacket and a series of valves for sampling (Fig. 24).^307^

**Fig. 25:**
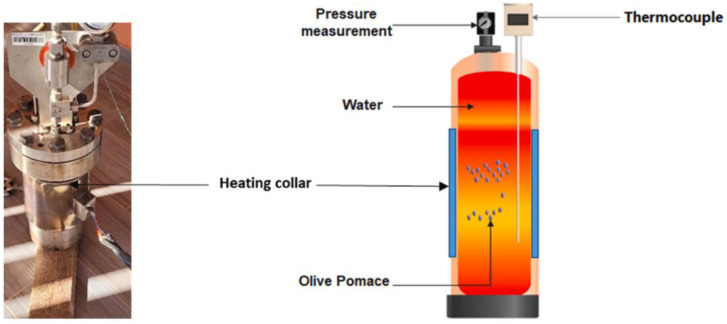
The heating collar encircling the reactor (left) and the batch reactor's components (right).^308^

A cutting-edge hydrothermal reactor system that combines a batch reactor with a solar-powered heating collar was designed (Fig. 25). At a temperature of 220 °C and pressure of 40 bar, this system was designed to reach the subcritical conditions required for hydrothermal carbonization. The device successfully heated the reactor using solar energy, indicating a viable strategy for sustained biomass conversion.^308^

**Fig. 26:**
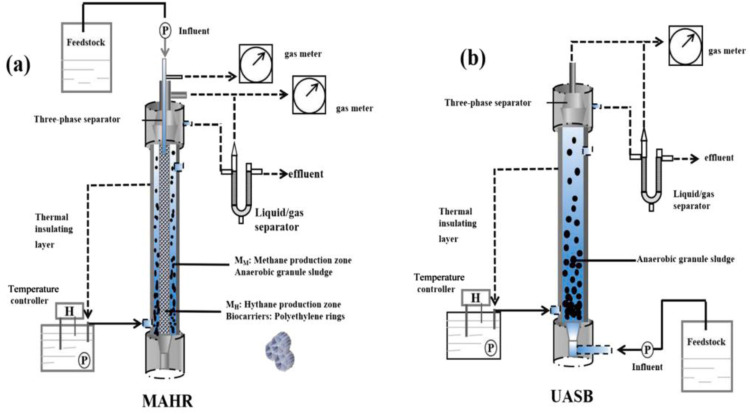
Schematic diagrams of MAHR (a) and UASB (b) for ongoing AD trials.^311^

A hydrothermal reactor system was developed consisting of a 500 mL and a 10 mL unit, each equipped with a band heater and a cylindrical steel block to ensure optimal thermal transitions. The device attained a heating rate of roughly 10 °C min^−1^ and had an internal coil for rapid cooling as well as a magnetic stirrer. Following the reaction, the solid–liquid mixture was filtered, and Soxhlet extraction with dichloromethane was used to extract the bio-crude product.^309^ Hydrothermal carbonization wastewater (HTCWW) was treated using a novel multistage anaerobic hythane reactor (MAHR). Operating with a hydraulic retention period of 12 h and an organic load rate of 20 g SCOD per L per day, this reactor was constructed with an external up-flow sludge bed and an interior downflow biofilter bed. The potential for anaerobic HTCWW valorization was indicated by the MAHR's successful COD removal and methane production.^310^ In addition, the performance of MAHR was compared with conventional up-flow anaerobic sludge bed reactor (UASB) by conducted continuous anaerobic digestion from microalgae hydrothermal carbonization wastewater (HTCWW). MAHR showed better of treatment performance and stability when synthetic wastewater was gradually replaced by HTCWW. It achieved an average methane production rate (MPR) of 7.0L/L/d, with methane content over 70%, and removed 84% of soluble chemical oxygen demand (SCOD) when HTCWW made up 100% of the organic loading rate (OLR) (Fig. 26).^311^

**Fig. 27:**
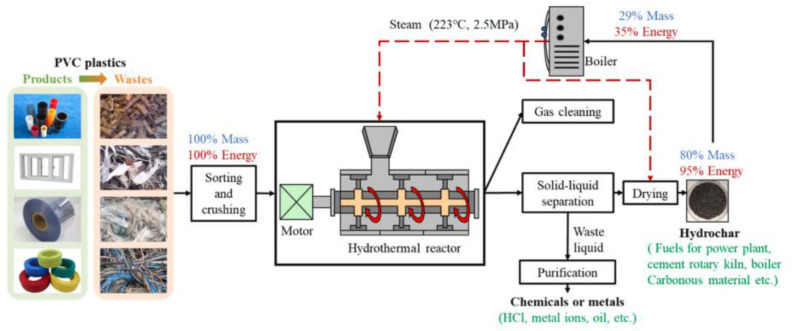
Hydrothermal PVC plastic treatment.^315^

The main techniques for evaluating the extraction of phenolic chemicals from black liquor are batch and continuous hydrothermal reactors. A 0.5 L continuous reactor from TOP Industries and a 0.6 L stainless-steel batch reactor from Parr Instruments were employed. The continuous reactor operated at a flow rate of 1.5 L h^−1^. Temperatures of 280 and 350 °C were applied to the reactors, and changes in pH affected the extraction effectiveness.^312^ A co-current flow configuration was employed in a continuous oscillatory baffled reactor (COBR), which had a heating jacket. Three heating jackets along the length of the reactor enabled perfect temperature control, which is extremely important for the quality of the final output. To effectively analyze the reactive suspension, thermocouples and sample points were used to monitor the system.^313^ A continuous flow reactor on a pilot scale was intended for the hydrothermal production of zeolite LTA. As it does not clog, which is a common problem with tubular reactors, this reactor effectively produces highly crystalline zeolite nanoparticles. Space time yields (STYs) ranged from approximately 700 to 1100 g L^−1^ h^−1^ when the system reached steady-state production rates of 55–80 g h^−1^ with a residence period of less than 10 min. To optimize the continuous flow hydrothermal process, a coupled CFD model with a population balance model was established, which closely matched the experimental data.^314^ A commercial-scale setup with a 5 m^3^ capacity, intended for the treatment of medical and municipal solid waste (MSW), was described using a hydrothermal reactor system. Its major components, which enable effective processing under high-pressure and -temperature conditions, include a steam boiler, reactor with a stirrer, condensation system, and automatic control system (Fig. 27).^315^

**Fig. 28:**
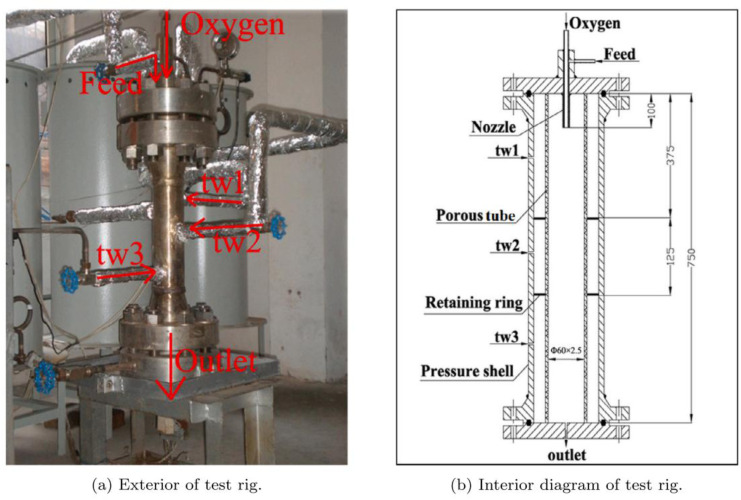
An experimental TWR setup featuring (a) exterior and (b) interior test-rig diagrams.^317^

A transpiring-wall reactor (TWR) efficiently treats organic wastewater using hydrothermal flames. By sustaining high temperatures (650–1200 °C) and limited residence times (10–100 ms) to prevent coking and plugging challenges, this system overcomes preheating difficulties and improves feed degradation. In supercritical water oxidation systems, the TWR prevents corrosion and salt deposition and has considerable economic utility, especially for wastewater with high salinity or solid content (Fig. 28).^316^

**Fig. 29:**
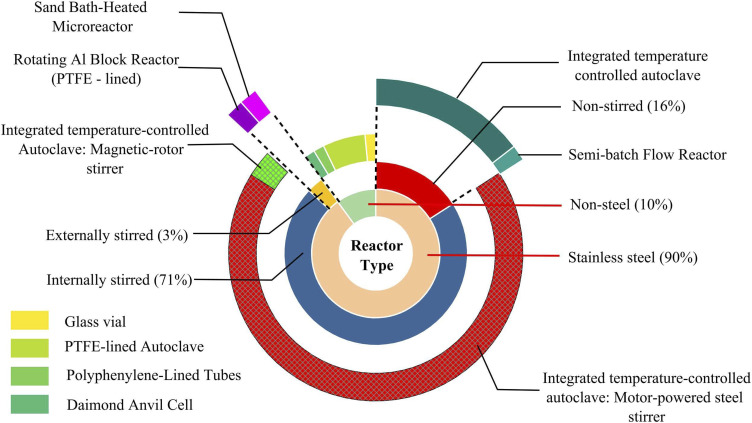
Typical reactors for PVC hydrothermal dechlorination on a benchtop scale.^318^

A Teflon-lined reactor that rotates has been used, which is especially designed to reduce metal contamination from steel parts. Typically, 0.10 g of PVC sample was dissolved in 10 mL of water and certain quantities of nickel and iron salts. Significant increases in dechlorination were noted at 210 °C, demonstrating the function of the reactor in promoting the reaction under mild conditions (Fig. 29). The reactor was maintained at a regulated temperature.^318^

**Fig. 30:**
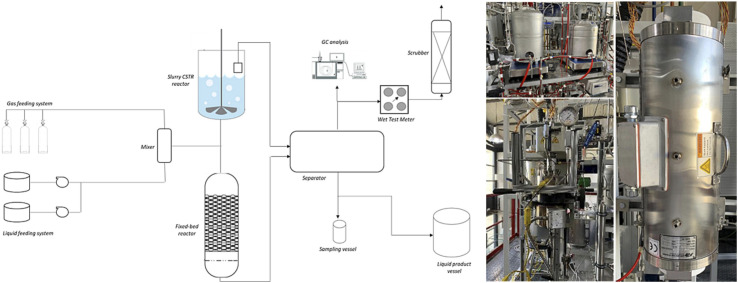
A simplified flow diagram of a hydrotreatment test unit.^319^

A flexible pilot-scale reactor system for the continuous catalytic hydrotreatment of hydrothermal liquefaction oil generated from sewage sludge was developed, which involves both slurry and fixed-bed reactors (Fig. 30). Furthermore, owing to the improved residence time and reduced mass-transfer constraints, the slurry reactor consistently outperformed the fixed-bed reactor in terms of heteroatom removal and cracking efficiency, resulting in a 35% decrease in the nitrogen content of the upgraded oil at 350 °C.^319^

A cavity receiver for radiative and thermal transfer phenomena and a pressurized mixing vessel for reactions have contributed to a hydrothermal reactor system. The system has a highly reflective cylindrical chamber that circulates a biomass-water suspension over a system of absorbing tubes. In addition to attaining thermal efficiencies above 80% and biogas and biocrude yields of approximately 20% and 30%, respectively, the design seeks to uniformize the dispersion of solar radiation while reducing thermal gradients and stresses.^320^ A continuous tubular HALT reactor was developed. The system utilized a positive displacement HPLC pump to deliver feedstock that had been premixed with NaOH at different concentrations, and it operated at a steady 350 °C. Products are collected post-quenching and throttled through a back-pressure regulator after the reactor is coiled and immersed in a hot sand bath.^321^ The pipe-in-pipe structure of a continuous-flow nozzle reactor combines cold biomass feed with hot, preheated compressed water at the nozzle outlet to achieve high heating rates. A high-pressure syringe pump provides the cold flow of lignosulfonate solution, whereas a high-pressure liquid chromatography pump regulates the hot water flow rate. The system operates at 350 °C and 21.4 MPa.^322^ In the hydrothermal reactor system, the working fluid is near-critical water in a cylindrical configuration. To explore natural convection under various heating schemes, the reactor sidewall was split into two halves with different heat flux densities. Operating at an initial temperature of 407 °C and a pressure of 25 MPa, the system focuses on how the heat flux ratio affects the flow structure, temperature distribution, and wall heat transfer during the feedstock preheating and gasification processes.^323^ Hydrothermal carbonization (HTC) is facilitated by heating compressed wet sewage sludge to a predefined hydrothermal reaction temperature in a hydrothermal reactor system. By effectively producing high-calorific-value hydrochar products using the released heat for preheating, this exothermic reaction reduces the need for hot primary air. Contrary to direct drying techniques, this process produces hydrochar with a lower moisture content, enhancing combustion performance. It also involves depressurization and water separation.^324^ A hydrothermal reactor system can treat sewage sludge by operating at high temperatures (180–260 °C) and pressures (10–50 bar). This method, called hydrothermal treatment (HT), efficiently breaks down microbial structures, improves dewatering efficiency, and reduces sludge cohesiveness. To enhance the biodegradation and energy recovery potential of wet digested sludge, which has a high water content of approximately 80%, the system transforms it into a solid substance termed hydrochar, which has reduced moisture and organic pollutants.^325^ A closed-system batch-type reactor was used to replicate the seawater conditions of the Precambrian period. It uses a titanium-headed gold bag for flexibility and resistance to high-temperature fluids and operates at 300 °C and 500 bar. To reproduce Precambrian high salinity and low sulfate levels, a mixture of synthetic Cl-rich seawater and powdered mid-ocean ridge basalt (MORB) was added to the reaction cell. Fluid samples were collected throughout the experiment to observe compositional changes.^326^ A 415 mL continuous stirred tank reactor (CSTR) and a 270 mL continuous plug flow reactor (PFR) were used. With this arrangement, biomass can be processed efficiently. Biocrude and other byproducts are produced by continuously pumping, heating, and subjecting the biomass slurry to high-pressure and -temperature conditions.^327^ Laser-microtextured Ti_6_Al_4_V systems were hydrothermally treated using distilled water alone, without the use of any chemical reagents, in a reactor that was heated to 180 °C. A bioactive and hydrophilic oxide layer was formed on the titanium surface during the three-hour treatment, which is essential for improving osseointegration in orthopedic implants.^328^ The high-pressure/high-temperature batch reactor used in hydrothermal desulfurization (HT-DS) was a Parr Instruments 4560 type from the USA. During the desulfurization process, this reactor, which has a 300 mL capacity, is used to convert dibenzyl sulfide (DBS) both catalytically and non-catalytically when heavy water (D_2_O) is present as a hydrogen donor.^329^

## 6 Optimization studies

### 6.1 Effect of time on methane oxidation

Time can affect methane oxidation by reducing conversion during oxygen-deficient phases as the oxygen reservoir from a Mn–Fe spinel layer is consumed. As a result, the CH_4_ conversion gradually decreases, whereas conversion peaks occur upon the reintroduction of oxygen.^330^ Methane oxidation is influenced by time because the total catalytic performance is affected when H_2_O is used as a diluent gas, as it minimizes the residence time of CH_4_/O_2_.^331^ Methane oxidation exhibits notable time-dependent behavior, particularly when oscillation events affect concentration determinations. The consumption of methane at 30 bar was negligible between 700 and 900 K.^332^ Methane oxidation is greatly influenced by reaction time; the yield of CH_3_OH is steady between 0 and 40 min, but it decreases between 40 and 60 min as a result of overoxidation, which yields byproducts such as CH_3_OOH and HCHO. Although the overall yield slightly decreases, longer reaction periods result in decreased CH_3_OH selectivity.^333^ A key variable in methane oxidation is time; prolonged rich phases delay the initiation of steam reforming and the complete oxidation of methane. When the catalyst state is more oxidized at 0.06*λ*Amp, dynamic CH_4_ reforming begins later, affecting the total conversion rates, whereas it starts earlier at 0.10*λ*Amp .^2^ Considering that the anaerobic oxidation of methane (AOM) increases slowly, time has an enormous effect on methane oxidation.^334^ A 300 W xenon lamp was used for illumination during the methane oxidation process for three hours. This had a significant effect on the reaction outcome and produced liquid oxidation products. To achieve high yields of products such as CH_3_OH and HCHO, reaction time optimization is essential.^335^ In contrast to other catalysts, the photocatalytic oxidation of methane to methanol exhibited notable time dependence, with methanol production reaching 355.25 mmol after 6 h. Only approximately 40% of the methanol had decomposed after a day, suggesting that production and degradation were balanced over time.^336^ The contact period has an immense effect on methane oxidation; shorter contact times (0.02 to 0.05 s) result in higher selectivity for partial oxidation products, confirming a direct reaction mechanism as opposed to reforming processes. This implies that lowering the residence period improves the methane oxidation efficiency.^337^ During methane oxidation, methanol selectivity is enhanced by prolonging the reaction duration. More specifically, the catalyst effectiveness is improved by incorporating mesopores and modifying the iron content, which eventually increases the methanol turnover frequency, particularly at moderate temperatures.^190^ In an AME-AD system, the methane oxidation rates were significantly affected by time. The oxidation rates progressively increased from 0 to 96 h, attaining their maximum at that time. However, the treatment process C5 continued to have the highest rate of oxidation until the end of the blooming period, but the rate of treatment for process C6 began to decrease thereafter.^338^ Time has an important effect on the production of methanol during methane oxidation, as indicated by a sudden increase in the peak intensity of CH_3_O species during the first two minutes of exposure, followed by a progressive increase over time.^339^ The stability of the CH_4_ conversion rates, which remained steady up to 415 °C, indicates the effect of time on methane oxidation and shows that dithering pulses have no effect on activity throughout this time. However, reflecting the differences in oxygen availability, conversion rates vary at higher temperatures.^340^ The peak linked to methane oxidation increased significantly when transitioning from air to methane, indicating that time permits methane oxidation reactions to become established and improves the overall performance.^341^

### 6.2 Effects of temperature on methane oxidation

Higher temperatures induced faster ignition and more CO production, which had a substantial impact on methane oxidation. Oxygenates such as DEE and DMM promote reactivity and minimize soot production in fuel-rich situations by shifting the ignition and soot generation to lower temperatures.^342^ At low temperatures (*T* < 800 K), methane oxidation depends mainly on O_2_ (^1^Δg) reactions; however, at higher temperatures, CH_3_O_2_ converts into a transient intermediate, which leads to different reaction pathways and dynamics in the oxidation process.^343^ Temperature has a significant effect on the oxidation of methane (CH_4_), as higher temperatures accelerate the reaction. While mass transfer is limited in the first phase of the catalyst process, when kinetics predominate, the temperature and concentration profiles remain flat. However, a hot patch forms on the catalyst surface as the reaction proceeds, indicating that temperature is an important factor in the entire oxidation process, especially as it approaches thermodynamic equilibrium.^344^ As shown by the higher activation energy of 57.9 kJ mol^−1^ for CH_4_ under photothermal conditions compared to 112.1 kJ mol^−1^ under thermal conditions, temperature has a significant impact on methane oxidation. This implies that light radiation effectively reduces the activation barrier for methane conversion.^345^ The appropriate temperature for 50% CH_4_ conversion decreased from 288 °C (without H_2_O) to 260 °C (with H_2_O), indicating that temperature has a significant effect on methane oxidation. CH_4_ conversion also occurs at 100 °C, indicating that higher temperatures enhance the gasification of carbonates produced during the reaction.^346^ The presence of hydrogen sulfide (H_2_S) causes the oxidation of methane (CH_4_) to begin earlier than it would otherwise. Temperature has a significant impact on this process. The addition of H_2_S specifically anticipates the initiation of CH_4_ oxidation by 40–75 K over a range of equivalency ratios, despite the fact that H_2_S itself begins to oxidize at temperatures as low as 650 K.^347^ The oxidation of methane is profoundly affected by temperature. The maximum yield and selectivity of methanol were measured at a reduction temperature of 300 °C; however, the methane conversion displayed very little variation as the temperature increased. Plasma catalysis is more efficient for direct oxidation under atmospheric conditions, as indicated by the low methanol production at high temperatures (500 °C), despite an increase in CH_4_ conversion.^339^ Up to 415 °C, the conversion of methane (CH_4_) remained steady, indicating a positive effect of temperature on the conversion process. Beyond this, oscillations in the formation of NO were noted at approximately 420 °C, but CH_4_ selective reduction (CH_4_-SR) occurred at 425–435 °C. When palladium (Pd) was partially reoxidized, the conversion was reduced, but complete NO reduction at high conversion regimes indicated an active CH_4_-SR.^340^ The most potent catalyst, Ce/CuCo/Al, achieved a “light off” temperature of 400 °C, confirming the strong temperature dependence of the oxidation of methane. The activity order below 300 °C is Gd/CuCo/Al > Ce/CuCo/Al > Nd/CuCo/Al; beyond this temperature, it changes to Ce/CuCo/Al > Nd/CuCo/Al > CuCo/Al.^348^ As the temperature rises during coal oxidative pyrolysis, methane (CH_4_) emission begins at approximately 300 °C, peaks at approximately 650 °C, and then progressively declines until 900 °C. This temperature-dependent trend demonstrates that the thermal parameters of the oxidative pyrolysis process have a major impact on methane production.^349^ Throughout the photocatalytic methane oxidation reaction, the temperature was maintained between 25 and 35 °C. Temperature control is essential for maximizing reaction conditions and product yield, as demonstrated by the fact that, during a 2-hour reaction, the temperature decreased below 10 °C to facilitate product collection and analysis.^350^ Pure methane starts to oxidize at temperatures higher than 1080 K, but adding 10% NH_3_ lowers the ignition point to approximately 970 K, increasing the reactivity by approximately 150 K. However, the ignition temperature rises as the NH_3_ concentration exceeds 40%, indicating that NH_3_'s stimulating influence on methane oxidation at higher temperatures declines.^351^ The CH_4_ oxidation activity of Pd-based catalysts decreases at high temperatures, particularly above 650 °C, where Pd does not chemisorb oxygen, resulting in substantial deactivation. However, Pt-based catalysts remain more stable under comparable circumstances, indicating that temperature is an important variable in the functioning of these catalysts during methane oxidation.^352^ Grassland and city park ecosystems exhibited the highest CH_4_ absorption at 22–23 °C, indicating that temperature has a substantial impact on methane oxidation in these ecosystems. Higher temperatures promote methane oxidation rates, especially in urban soils during warmer months, as proven by the yearly mean *Q*_10_ values for CH_4_, which varied from 1.26 to 1.49.^353^ Methane (CH_4_) oxidation increases dramatically with temperature, particularly in high-temperature areas where non-selective oxidation is more prominent. NO_*x*_ conversion is negatively affected by the competition between the SCR process and CH_4_ non-selective oxidation, particularly above 600 °C.^354^ The production of C_1_ oxygenated products is enhanced by increased temperature, which has a substantial impact on methane oxidation. When compared to lower temperatures, the overall turnover frequency of C_1_ products within the catalyst at 70 °C was 8380 h^−1^, indicating a significant increase in activity.^355^ In the direct oxidation of methane (CH_4_), the performance of Fe_2_O_3_-c nanocrystals is strongly influenced by the reaction temperature. In particular, under ideal circumstances at 100 °C, high selectivity of 89.4% and a production rate of 6.315 mmol g_cat_^−1^ h^−1^ for HCOOH are achieved.^356^ Temperature is an important variable in methane oxidation; high temperatures may prevent methanotrophic activity.^357^ The methanol turnover frequency (TOF) during methane oxidation was improved by increasing the temperature, and significant increases were observed at temperatures above 320 °C. For example, the TOF values more than doubled at 340 °C compared to those at lower temperatures, showing enhanced reaction efficiency.^190^ Increasing the starting temperature promotes reactions that generate O and OH radicals, increasing the flammable range and enabling flame propagation, especially at lower flammability limits (LFLs), which improves methane oxidation.^358^ The rate of methane oxidation is extremely dependent on temperature, with higher rates observed at higher temperatures. Directly correlated with microbial activity, the methane oxidation potential (MOP) is exceptionally low below 10 °C and significantly increases at temperatures up to 35 °C.^359^

### 6.3 Effects of pressure on methane oxidation

At high pressures (>10 bar), the type of inert gas utilized affects the frequency at which methane oxidizes, with substantial differences in the conversion rates recorded. In particular, lighter noble gases, such as helium, exhibit lower conversion rates, whereas heavier gases, such as xenon, exhibit the fastest methane pyrolysis.^360^ Methane oxidation is greatly influenced by pressure; when pressure rises from 1 to 90 atm, the onset consumption temperature drops from 1075 to 700 K. Likewise, when pressure is above 30 atm, NTC behavior is observed at higher pressures, especially between 810 and 900 K.^361^ While achieving selectivity of up to 90.7%, increasing O_2_ pressure increases the CH_3_OH yield by rapidly producing ˙CH_3_ and H_2_O_2_. Pressures exceeding 2.0 bar, however, lead to reduced selection due to the generation of byproducts such as CH_3_OOH and HCHO, while excessive CH_4_ pressure also decreases selectivity because of increased CH_3_ production.^333^ Pressure promotes the production of soot precursors *via* efficient pressure-dependent processes, and temperature increases soot loading.^362^ Methane (CH_4_) permeability is substantially decreased by feed pressure as pressure increases, with the loss in CH_4_ permeability being lower in magnitude (14%) than that of H_2_, which decreases by 39%, when pressure increases from 1 to 4 bar.^363^ In methane catalytic partial oxidation (CPO), increasing pressure leads to an increase in the output temperature, whereas CH_4_ conversion and syngas selectivity decrease. However, mass transport constraints mostly overlook kinetics, with pressure predominantly affecting thermodynamic behavior.^344^ As the pressure increases, CH_4_ is more easily dissolved in water, increasing the amount of CH_3_OH produced during photocatalytic oxidation. According to this relationship, the efficiency of an AC-Co_1_/PCN KOH photocatalyst in converting methane to methanol is positively impacted by increased pressure.^336^ By lowering the initial temperature and increasing the frequency of the reactions between CH_4_ and NO_2_, higher reaction pressures promoted methane conversion. It was determined that 1.0 MPa was the ideal pressure for producing methanol, and that the selectivity for methanol peaked at higher pressures before slightly diminishing.^364^ Prior to ignition initiating combustion, conditions that correspond closely to those found in technical combustion systems, such as gas turbines and internal combustion engines, can be observed mainly due to the elevated pressure of 6 bar during methane oxidation.^365^ Under stoichiometric and oxidizing conditions, the initial temperature for methane oxidation was significantly reduced as the pressure increased. The addition of NO_*x*_ intensifies this effect, establishing an intimate link between the oxidation efficiency and pressure.^366^ The effectiveness of CH_4_ extraction is improved by a higher injection pressure, which enhances the competitive adsorption effect. However, higher pressures also cause coal to expand, which lowers permeability and may make it more difficult to extract CH_4_.^367^ The laminar burning velocity (LBV) of methane decreases with increasing pressure, primarily because high pressure lowers the rates at which NH_2_ is converted to HNO and H radicals by approximately 34.2% and 29%, respectively.^368^ Hydrogen sulfide (H_2_S) can promote the immediate oxidation of CH_4_, but methane (CH_4_) takes longer to oxidize at lower temperatures under high pressure. However, CH_4_ delays H_2_S oxidation under ambient conditions.^347^ The conditions for methane oxidation were optimal at a capillary pressure of approximately 500 hPa. There was an approximately 50% decrease in the oxidation rate when the soil moisture decreased below 7000 hPa. Methane oxidation is expected to be aided by an increase in pore capacity at 500 hPa.^369^ Increasing the pressure promoted the consumption of reactive species, which improved methane oxidation and lowered NO emissions. At medium ammonia content, where the pressure increases the overall reaction rate, especially around *X*_NH_3__ = 0.5 for NH_3_/CH_4_/air flames, the ideal conditions for NO reduction are met.^370^ Significant CH_4_ consumption originates from the increased reactivity of the methane oxidation system due to the pressure increase. However, NH_3_ consumption is more strongly hindered by CH_4_ than by H_2_, particularly at lower temperatures, where reactive radicals are less prevalent.^332^ During methane oxidation, elevating the N_2_O pressure from 10 kPa to 30 kPa caused overoxidation, which reduced the methanol selectivity from 31% to 20% due to the formation of unwanted byproducts.^190^ The yield of C_1_ oxygenates was significantly increased while maintaining high selectivity by increasing the partial pressure of CH_4_ from 0.5 MPa to 2 MPa, revealing methane-limited functioning during the reaction. Moreover, as the O_2_ pressure increased, the yield of C_1_ oxygenates also increased, demonstrating its crucial function in the photocatalytic oxidation process.^350^

### 6.4 Effects of pH on methane oxidation

High pH levels are occasionally harmful or interfere with cells mediating anaerobic oxidation of methane-sulfate reduction (AOM-SR) processes; they can also increase alkalinity and hydrogen sulfide products, and may adversely influence the efficiency of methane oxidation.^371^ pH affects methane anaerobic oxidation because alkaline conditions enable carbonate minerals to develop through reactions between bicarbonate and calcium ions, increasing the effectiveness of methane oxidation.^372^ Typically, anaerobic oxidation of methane (AOM) microbes favor a pH range of 7.0–7.4, with soils below 20 cm depth being neutral at pH 7.1, which promotes AOM activity.^373^ The relative insensitivity of aerobic methanotrophs to variations in pH indicates that their activity in methane oxidation pathways is not significantly altered by pH changes.^374^ Sediment pH and the dynamics of anaerobic oxidation of methane (AOM) activity are strongly correlated, indicating that variations in pH may substantially affect the rates of methane oxidation in riverine sediments.^375^ Methanotroph activity is inhibited by low pH in acidic soils, which could adversely influence methane oxidation processes and decrease the overall number of these microorganisms necessary for transforming methane into more harmless forms.^376^ Methanotrophs are adversely affected by low soil pH, with their activity inhibited in acidic soils. Methane oxidation dynamics in soil are significantly affected by pH and redox conditions, as shown by the fact that type-I methanotrophs proliferate more easily than type-II methanotrophs when the soil redox potential increases.^377^ Appropriate pH levels can improve the attachment of microbes to microplastics, which in turn affects their metabolic processes, such as methane oxidation. However, under certain conditions, precise pH values and their direct effects on methane oxidation rates have not been explained.^1^ By facilitating microbial respiration and increasing the variety of methanotrophic bacteria, particularly *Methylosinus* and *Methylocystis*, which are essential for soil methane consumption, increasing the pH of the soil improves methane oxidation. Higher pH values also provide a favorable environment for methane-oxidizing bacteria, encouraging more vigorous oxidation.^378^ Methane oxidation is influenced by pH; acidic soils have higher levels of pmoA genes, which increase methanotrophic activity. Conversely, neutral soils had minimal effects on functional genes and CH_4_ emissions, indicating that pH is a key factor in methane oxidation efficiency across soil types.^379^

### 6.5 Effects of the amount of water on methane oxidation

The role of water as a “soft” oxidant greatly improves the selectivity of methane oxidation for methanol by promoting high selectivity in its production and accelerating methanol desorption. However, there are still challenges with catalytic systems as a whole with respect to useful applications.^380^ In the photocatalytic oxidation of methane, water serves as the sole oxidant, which accelerates the conversion process. During the reaction, the yields of carbon monoxide and other products increased significantly in the presence of water.^284^ Water promotes the oxidation of methane by participating in subsequent processes, primarily steam reforming, which contributes to the overall methane oxidation process and affects product selectivity.^381^ Methane oxidation is influenced by the amount of water provided; when the water vapor flow rate increases, there is a change in the reaction dynamics.^382^ During tidal inundation, the presence of overlying water decreased methane oxidation rates when methanotrophic activity was limited by lower dissolved oxygen levels. In contrast, sediment exposed to air reduced methane flux by approximately 62% and increased methane consumption by approximately 227% compared to inundated settings.^383^ Methane oxidation is hindered by water, and its inhibitory effects increase with concentration. In particular, the catalytic activity still exhibits a volcano-shaped dependence on the silica-to-alumina ratio (SAR) even when there is only 8% water in the feed.^384^ For both AuPd and AuPd–CuO_*x*_ catalysts, the presence of water reduces the methane oxidation rates by approximately a factor of two, showing that water precludes methane combustion according to the conditions under consideration.^385^ The oxidation rates depend on the methane concentration of the electrolyte, with water serving as a channel for the production of radicals. Methane may become less concentrated locally, and effective oxidation close to the cathode may be impeded by surplus water.^386^ By controlling the temperature and minimizing soot production during methane oxidation, the amount of water fed to a partial oxidation reactor can improve the overall efficiency of the reaction.^387^ Shorter water retention periods may improve methane oxidation efficiency in these systems, as indicated by the decrease in methane emission from artificial wetlands as the hydraulic retention time (HRT) decreased.^388^ Water has two main adverse effects on methane oxidation: it causes a large loss of catalytic activity, especially during the cooling phase, and it moves the conversion curves to higher temperatures. Changes in the oxidation state of palladium and competing adsorption with methane are responsible for water-induced deactivation.^389^ Water was shown to have a negative impact on catalytic activity because it significantly delayed methane oxidation, raising the temperature required for 50% conversion by approximately 50 °C. Methane and water compete for active sites, thereby contaminating the catalyst surface.^390^ When water vapor is introduced to the reactor after the methane has been turned off, it improves the oxidation reaction of methane and causes mNi/Ce_0.8_Zr_0.2_O_2_ to undergo oxidation efficiently until the hydrogen concentration declines to zero.^391^ In the oxidative coupling of methane (OCM), a considerable increase in water vapor pressure (*P*_H_2_O_) promotes the production of hydrogen and ethane by improving methane oxidation. Water plays a critical role in the reaction, and at high *P*_CH_4__ (200 kPa), ethane production increases with *P*_H_2_O_, exceeding 65% selectivity at *P*_H_2_O_ = 3 kPa.^392^ Methane conversion is enhanced by increasing the water content, but the C_2_-hydrocarbon selectivity seems unaltered. Higher water content also boosts the output of C_2_-hydrocarbons.^393^ Over oxide-supported Pd catalysts, methane oxidation is inhibited by increasing the amount of water vapor. More specifically, Pd/Al_2_O_3_ exhibited a substantial reduction in activity at a mere 1 vol% water, but Pd/SnO_2_ and Pd/Al_2_O_3_–36NiO exhibited nearly constant activity at elevated concentrations.^394^ The catalytic characteristics of Pd-containing catalysts are decreased by the presence of water vapor in the feed, which affects the rates at which they function in methane oxidation.^395^ Water vapor lowers the conversion rates by having a major effect on methane oxidation activity. In contrast to those without barium (Ba), catalysts containing Ba exhibit enhanced resistance to water deactivation, sustaining higher activity levels, particularly after regeneration at high temperatures.^396^ The rate of methane oxidation is greatly affected by changes in water level; it is maximum during dry periods (35.69 to 56.32 nmol per (g soil) per day) and lower during deluges (11.58 to 11.98 nmol per (g soil) per day).^397^ Water inhibits methane oxidation and significantly reduces the catalytic activity, particularly in a 0.4Pd/CeO_2_ catalyst by preventing oxygen spillover between Pd and CeO_2_. Samples with high loading (2.0 wt% and 4.0 wt%) displayed better water resistance and recoverable activity after water removal.^398^ The amount of steam influences methane oxidation, and a higher steam flow increases the rate of hydrogen generation. Thus, when nitrogen propelled steam into a membrane reactor, the highest rate of hydrogen synthesis was 3.7 mL min^−1^ cm^−2^.^399^ Methane (CH_3_) oxidation is strongly influenced by the water content of landfill cover soil. Although soil water potential is a more accurate indicator that closely corresponds to field capacity, the ideal gravimetric water content for CH_4_ oxidation ranges from 15% to 25%. CH_4_ oxidation rates can be considerably lowered by either excess or insufficient water.^400^

## 7 Conclusions

The challenge of efficiently and selectively converting methane into methanol remains a significant hurdle in the field of catalytic valorization. This review emphasizes the notable advancements in catalytic approaches, with a focus on the growing importance of MOF-based catalysts. These catalysts offer adjustable porosity, large surface areas, and the potential for the precise engineering of active sites. The incorporation of nanoparticles, such as in monometallic and bimetallic NP@MOF catalysts, has led to promising improvements in catalytic performance, especially regarding selectivity and stability. Traditional supported catalysts, including those based on noble and transition metals, continue to be crucial in methane oxidation, with ongoing efforts to enhance their efficiency and cost effectiveness. The choice of oxidant, particularly in oxygen-based systems, greatly affects methanol yield and selectivity, requiring careful optimization of the reaction conditions. Additionally, reactor design is vital for process scalability, with hydrothermal reactor systems emerging as a promising technology for methane oxidation under mild conditions. Optimization studies have shown that parameters like temperature, pressure, pH, and water content directly impact methane conversion efficiency and methanol selectivity. However, achieving industrial-scale application necessitates further improvements in catalyst stability, cost effectiveness, and energy efficiency. Future research should aim to develop more robust and highly selective catalytic systems, explore alternative oxidants, and optimize reactor configurations to enhance process sustainability. The integration of computational modeling and advanced characterization techniques will be essential for understanding reaction mechanisms and designing next-generation catalysts. By addressing these challenges, the conversion of methane to methanol can be further refined to support sustainable energy solutions and carbon management strategies.

## Author contributions

U. Zayyanu Gwandu: writing – original draft; G. Mohammad-Alsultan: review & editing, G. Abdulkareem-Alsultan: writing – original draft, writing – review & editing, supervision; N. Asikin-Mijan: supervision; H. V. Lee: writing – review & editing; and Yun Hin Taufiq-Yap: supervision, funding acquisition.

## Conflicts of interest

We declare that we have no significant competing financial, professional or personal interests that might have influenced the performance or presentation of the work described in the paper.

## Data Availability

No primary research results, software or code have been included and no new data were generated or analysed as part of this review.
